# Investigating developmental changes in scalp-to-cortex correspondence using diffuse optical tomography sensitivity in infancy

**DOI:** 10.1117/1.NPh.8.3.035003

**Published:** 2021-07-24

**Authors:** Xiaoxue Fu, John E. Richards

**Affiliations:** University of South Carolina, Department of Psychology, Columbia, South Carolina, United States

**Keywords:** diffuse optical tomography, near-infrared light spectroscopy, Monte Carlo simulation, infant, development, head models

## Abstract

**Significance:** Diffuse optical tomography (DOT) uses near-infrared light spectroscopy (NIRS) to measure changes in cerebral hemoglobin concentration. Anatomical interpretations of NIRS data require accurate descriptions of the cranio-cerebral relations and DOT sensitivity to the underlying cortical structures. Such information is limited for pediatric populations because they undergo rapid head and brain development.

**Aim:** We aim to investigate age-related differences in scalp-to-cortex distance and mapping between scalp locations and cortical regions of interest (ROIs) among infants (2 weeks to 24 months with narrow age bins), children (4 and 12 years), and adults (20 to 24 years).

**Approach:** We used spatial scalp projection and photon propagation simulation methods with age-matched realistic head models based on MRIs.

**Results:** There were age-group differences in the scalp-to-cortex distances in infancy. The developmental increase was magnified in children and adults. There were systematic age-related differences in the probabilistic mappings between scalp locations and cortical ROIs.

**Conclusions:** Our findings have important implications in the design of sensor placement and making anatomical interpretations in NIRS and fNIRS research. Age-appropriate, realistic head models should be used to provide anatomical guidance for standalone DOT data in infants.

## 1 Introduction

Diffuse optical tomography (DOT) uses near-infrared light spectroscopy (NIRS) to measure changes in cerebral hemoglobin concentration.^1^^–^^3^ DOT does not provide anatomical information about the location of the hemodynamic signal. Spatial scalp projection can be implemented to interrogate the brain region(s) underlying the scalp sensor (i.e., optodes) locations.^4^ An alternative approach is to generate a forward model of DOT sensitivity using photon propagation simulations.^5^ The forward model can then guide DOT image reconstruction to recover the brain locations of hemoglobin concentration changes.^6^^,^^7^ There are considerable brain structural changes during infancy through childhood and adulthood.^8^^,^^9^ Accurate models of DOT sensitivity must account for age-related changes in the head.^10^

### 1.1 Scalp-to-Cortex Distances Measured by Scalp Projection

The initial step to understanding the underlying cortical structures being measured with NIRS is to measure the distance from the NIRS recording locations to the cortical surface. The 10–20, 10–10, and 10–5 systems provide a standardized and reproducible method for NIRS optode placement.^11^ Spatial scalp projection uses an algorithm to project a standard electrode location on the scalp down to a location on the cortical surface.^4^^,^^12^ Hence, it maps the scalp-to-cortex correspondence based on spatial locations. Adult studies projected 10–20^13^ and 10–10 electrodes^14^ to standard brain templates. Okamoto et al.^13^ found that the scalp-to-cortex distance was shallower in the frontal, temporal, and occipital regions but deeper in the parietal and along the intrahemispherical fissure. Scalp projection facilitates the understanding of the sampling depth required to measure the cortical activities using NIRS recording or DOT.

It is important to examine age-related changes in scalp-to-cortex distance. There is extensive brain morphological development from infancy to adolescence.^8^^,^^9^^,^^15^^,^^16^ One change is the distance from the scalp to cortical landmarks [Heschl’s gyrus, inferior frontal gyrus, frontal pole, occipital pole, parieto-occipital sulcus, and vertex (Vz)]. There are significant increases in scalp-to-cortex distances from newborn to age 12.^17^ Kabdebon et al.^18^ virtually placed 10–20 electrodes on individual MRIs from 3- to 17-week-old infants. The distance between the scalp electrode positions and the cortical surface decreased from the frontal to occipital locations. The pattern was not observed in adult head models.^13^ Studies adopting the scalp projection method indicate that the scalp-to-cortex distance is smaller in infants^18^^,^^19^ and children^20^ than in adults.^13^ Existing infant studies sampled a single age or a narrow age range. Hence, there is insufficient information about the differences across age on overall scalp-to-cortex distance or scalp-to-cortex distance by electrode positions.

### 1.2 Scalp-Location-to-ROI Mapping Using Scalp Projection

The scalp projection method can also be used to identify the correspondence between scalp electrode locations and the underlying anatomical regions of interest (ROIs). The electrode-location-to-ROI mapping can be established first by transforming the individual’s own MRI and electrode locations to a canonical average MRI template which has an anatomical stereotaxic atlas. Scalp electrode locations can then be projected to the cortical surface to identify the corresponding ROI(s). Adult studies demonstrated that there was a relatively consistent correspondence between the 10–20^13^ and 10–10^14^ electrode positions and the underlying macroanatomical ROIs. A methodological challenge in the ROI mapping is the limited availability of subjects’ own MRIs. Coregistration methods for standalone DOT data have been developed so that electrode positions on the subject’s scalp can be transformed to the space of reference MRIs in a database.^21^^,^^22^ The coregistration methods result in similar results as using subjects’ own MRIs.^4^^,^^22^

Coregistering scalp electrode locations with reference MRIs is an effective method for identifying electrode-location-to-ROI correspondence in infants. One method is to project individuals’ electrode locations to a template brain with ROI parcellations constructed from a representative infant or an average template based on infant MRIs. Kabdebon et al.^18^ transformed infants’ (3 to 17 weeks) individual head models to a 7-week-old infant template and found stable correspondence between 10–20 electrode locations and underlying microanatomical ROIs. Electrodes O1, O2, T5, and T6 were projected to more inferior cortical regions compared with the projection regions (left and right middle occipital and middle temporal regions) reported by Okamoto et al.^13^ This study showed that age-related changes in the head and brain structures may lead to age-related variations in the correspondence between electrode positions and brain structures. Tsuzuki et al.^23^ transformed macroanatomical landmarks identified in 14 3- to 22-month-olds’ MRIs to a 12-month-old template^18^^,^^23^^,^^24^ in reference to virtual 10–10 electrode locations.^11^ They found intersubject variations in the relative positions among the microanatomical landmarks. However, the differences were smaller than the region defined by the 10–10 electrodes. Hence, there may be relatively stable electrode-location-to-ROI correspondence across ages. One important limitation of these studies is the use of a single individual or single-age template as a reference for age ranges across which significant brain development occurs (e.g., 3 to 17 weeks; or 3 to 22 months).

The use of individual MRIs or age-appropriate MRI templates and stereotaxic atlases for standalone DOT data can reduce errors in coregistration and provide more accurate representations of electrode-location-to-ROI correspondence. Lloyd-Fox et al.^25^ coregistered channel locations (midway of between adjacent optode/electrode locations) on both the infants’ (4.5- and 6-month-olds) own MRIs and the age-appropriate average templates from the Neurodevelopmental MRI Database.^26^^–^^29^ Anatomical stereotaxic atlases were constructed for individuals’ own MRIs and age-appropriate average templates.^30^ The correspondence between channel locations and ROIs in individual MRIs was highly comparable with the correspondence identified in the age-appropriate average MRI templates. An alternative to coregistering with the subjects’ own MRIs or average templates is to use an MRI from another infant with a similar head size and age. A series of functional near-infrared spectroscopy (fNIRS) studies adopted Lloyd-Fox et al.’s^25^ coregistration method and positioned optode locations on close-head-size individual MRIs^31^ or both close MRIs and age-appropriate average templates.^19^^,^^32^^,^^33^ Scalp projection was used to establish probabilistic mappings between channel locations and atlas ROIs. The channels with a large probability of localizing to a target ROI were used for group-level analyses.

### 1.3 Use of DOT Sensitivity to Describe Scalp-to-Cortex Correspondence

The use of scalp projection for determining electrode-location-to-ROI correspondence is limited. It does not consider the interaction between near-infrared light and the optical properties of the biological tissues. Therefore, the approach is limited to spatially contiguous anatomical areas and cannot directly model the extent of the cortical regions being measured by NIRS recording nor the intensity of the signal generated by blood flow. It is possible that spatial projection from channel location maps to a different ROI than the channel of photons traveling from the source to the detector through the head and brain. An alternative to the scalp projection is to use DOT sensitivity patterns to determine the correspondence between scalp recording locations and underlying cortical anatomy. DOT sensitivity represents the extent to which the DOT instrument can detect changes in brain activities in the region that it is sampling.^34^ DOT sensitivity can be quantified by the probability of a photon traveling from a source-optode location to a voxel inside the head (2-point Green’s function).^35^ We will call this the “direct DOT.” A second approach to quantify DOT sensitivity in NIRS source–detector recording by calculating the product of the fluence distribution at the source location and the detector location [photon measurement density function (PMDF);^10^ 3-point Green’s function].^35^^,^^36^ We will call this the “source–detector channel DOT” and “S-D channel DOT.”^37^

The DOT sensitivity provides a measure of the scalp-location-to-ROI correspondence. NIRS channel location is defined as a point equidistant from the source and detector optodes. The direct DOT and S-D channel DOT fluence distributions can be used to estimate the distance from scalp locations to the surface of cortical regions that are measurable with DOT and localize the ROIs that would be sampled with NIRS.^38^ Studies estimated DOT sensitivity in head models with atlas parcellations in infants,^39^^–^^41^ children,^20^ and adults.^42^ These studies computed the channel-to-ROI correspondence and generated look-up tables to show channel-to-ROI probabilities. Zimeo Morais et al.^42^ computed the specificity of each channel to the corresponding ROIs using the S-D channel DOT measure. Such look-up tables facilitate the optimization of channel array design to maximize DOT sensitivity to user-specified ROIs^42^^,^^43^ and help to localize the possible ROIs that generated the fNIRS signals.^39^^–^^41^

### 1.4 Present Study

This study examined age-related changes in scalp-to-brain distance and the correspondence between scalp locations and anatomical regions. Our primary contribution was to use existing methods for DOT sensitivity with age-appropriate, realistic head models that cover the infancy period (2 weeks to 2 years) with narrow age bands and compare these to older children (4 and 12 years) and adults (20 to 24 years). Our choices of age groups reflect the primary aim of this study of examining age-related differences throughout infancy. The child and adult age groups served as “reference” groups for delineating how scalp-to-cortex correspondence in infancy may differ from the patterns in childhood and adulthood.

We employed different methods for assessing scalp-to-cortex correspondence. The scalp projection was used to estimate the distances between scalp electrode and channel locations and the cortical surface. We projected the electrode location to atlas locations delineated on individual MRIs to identify the anatomical mapping between scalp and ROI locations. We also computed the distance between scalp locations and the cortical locations with maximum direct DOT and S-D channel DOT fluence. The DOT sensitivity methods extend from the scalp project method by modeling how light from the source optode travels through the head and brain. We use the name “electrode” and “channel” interchangeably, though the “electrode location” properly refers to the scalp projection method whereas the “channel location” properly refers to the S-D channel DOT method. We provided look-up tables to present the probabilistic mapping between scalp locations and the underlying anatomical ROIs. Accurate and age-specific descriptions of scalp-to-cortex distance and scalp-to-ROI mapping are the bases for designing developmentally sensitive channel placements for NIRS recordings. Our look-up tables also provide important references for researchers to make anatomical interpretations of NIRS results from their specific age groups.

## 2 Method

### 2.1 Participants

The participants were 1058 typically developing participants ranging from 2 weeks to 24 years of age. The same sample was studied in Ref. 37. The MRIs were obtained from open-access databases and a local scanning facility. The sample consisted of nine participants (four females) from the Autism Brain Imaging Data Exchange (ABIDE),^44^ 280 (143 females) from the Baby Connectome Project (BCP),^45^ 177 (93 females) from the Early Brain Development Study (EBDS),^46^ 282 (106 females) from the Infant Brain Imaging Study (IBIS),^47^ 14 (5 females) from the Pediatric Imaging, Neurocognition, and Genetics Data Repository (PING),^48^ and 296 scans (141 females) from data collected at the McCausland Center of Brain Imaging (MCBI) or drawn from collaborative studies at other sites. Table 1 presents the number of MRIs for the open-access databases, separately for age and gender. The sample ages were narrowest in the infancy period (2 weeks, 1-, 1.5-, 3-, or 6-month intervals from 2 weeks to 2 years) and included exemplar ages in children and adolescent ages (4 and 12 years) and adult (20 to 24 years). All studies had institutional review board approval and informed consent. The University of South Carolina Institutional Review Board approved data collection at the MacCausland Center for Brain Imaging (MCBI) and the use of data from all open-access databases.

**Table 1:** Demographical information of study participants by age group, sex, and data source.

Participant information			Data source					
Age group	Total N	Female N	ABIDE N	BCP N	EBDS N	IBIS N	MCBI and collaboration sites N	PING N
2 weeks	41	24	0	3	38	0	0	0
1 month	96	40	0	17	79	0	0	0
2 months	68	40	0	8	60	0	0	0
3 months	38	21	0	24	0	0	14	0
4.5 months	54	29	0	41	0	0	13	0
6 months	74	35	0	0	0	60	14	0
7.5 months	61	17	0	0	0	49	12	0
9 months	60	35	0	48	0	3	9	0
10.5 months	42	21	0	40	0	0	2	0
12 months	109	36	0	0	0	89	20	0
15 months	78	41	0	63	0	8	7	0
18 months	76	31	0	36	0	8	32	0
2 years	66	22	0	0	0	65	1	0
4 years	24	9	0	0	0	0	10	14
12 years	37	14	9	0	0	0	28	0
20 to 24 years	134	77	0	0	0	0	134	0

Note: ABIDE, Autism Brain Imaging Data Exchange; BCP, Baby Connectome Project; EBDS, Early Brain Development Study; IBIS, Infant Brain Imaging Study; MCBI, McCausland Center for Brain Imaging; PING, Pediatric Imaging, Neurocognition, and Genetics Data Repository.

### 2.2 MRI Sequences

This study utilized T1-weighted (T1W) and T2-weighted (T2W) scans from each collection site. An age-appropriate average template was constructed for each age group. Details of the MRI acquisition protocols have been described in the literature on the Neurodevelopmental MRI Database.^26^^–^^30^^,^^49^ Details of the average template construction are also available in the literature and the Supplemental Material. All MRIs were converted to NIFTI compressed format with 32-bit floating point resolution. Data quality control and harmonization procedures were taken to ensure standardization among scans from different sites detailed in Refs. 27, 28, 29, and 49. MRI intensity variations found in different datasets were corrected. Bias-field inhomogeneity correction (N4 algorithm) was performed on the extracted T1W images.^50^^,^^51^ In addition, the peak of the gray matter (GM) intensity was normed to 100 to allow the scans from different sources to be standardized to the same voxel value range and resolution. All scans were visually inspected to ensure that there were no abnormalities in movement, image intensity, image orientation, or other artifacts.

### 2.3 MRI Preprocessing and Segmentation

First, the brains were extracted from the whole-head MRI volume in a procedure adapted from the FSL VBM pipeline.^52^ The T1W volume for each participant was registered to an age-appropriate average MRI template. The MRI templates came from the Neurodevelopmental MRI Database.^27^^–^^29^ The brain from the average template was transformed into the participant MRI space and used a mask on the head volume. The extracted masked data were then used with the FSL brain extraction tool program.^53^^,^^54^ Each brain was visually inspected and manually modified if necessary.

**Fig. 1:**
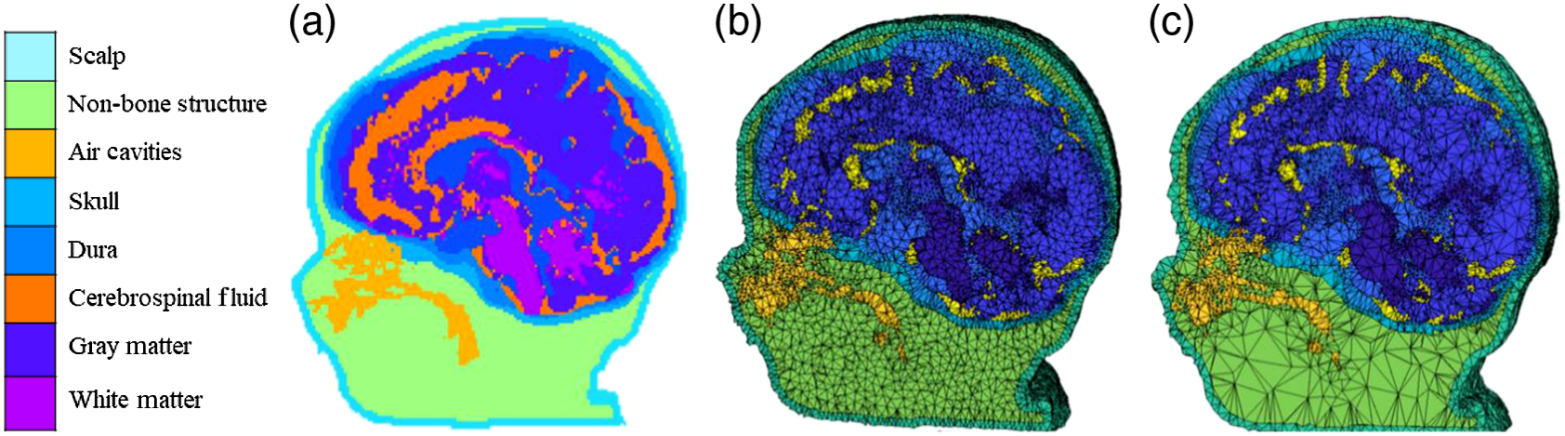
Segmented head MRI volumes. The examples were taken from the same 3-month-old infant MRI. (a) The segmented head model. Aqua is the scalp, green is the nonbone structure (muscle, sinew, fat), gold is the nasal and mouth air cavities, turquoise is the skull, light blue is the dura, orange is the CSF, dark blue is the GM, and purple is the WM. (b) The segmented head model with dense finite element mesh. (c) The segmented head model with sparse finite element mesh.

Second, each head MRI volume was segmented into 9 or 10 media types: GM, white matter (WM), cerebrospinal fluid (CSF), nonmyelinated WM, other brain matter, skin, skull, air, eyes, and other inside skull materials. Optical properties would need to be set for each of the tissue types for Monte Carlo simulations described below. The FSL FAST procedure^55^ was used to segment the T1W images into GM, WM, or other matter (OM). The tentative GM/WM classification from the initial step is problematic for infants at 12 months of age or younger whose brain lacks myelination (both GM and nonmyelinated tissue types appear dark in T1W).^8^^,^^28^ For infants in these age groups, the pattern of GM/WM in the 2-year-old average MRI template was used as a probability map to distinguish nonmyelinated tissues that should later be myelinated axons (WM), those that were GM or other nonmyelinated tissue. The CSF was identified in the T2W images using a threshold procedure. The CSF was removed from the materials from the FAST procedure, with the remainder defined as GM, WM, nonmyelinated WM, or other inside skull materials. The BETSURF procedure^53^^,^^54^ was used with the extracted brain, T1W and T2W volumes, to identify skull and scalp regions. The nasal cavity and eyes were identified manually using MRIcron.^56^^,^^57^ Finally, any OM inside the head volume not defined as above was defined as “other inside skull material.” This generally was in the region of the neck and consisted primarily of muscle and secondarily of spinal bone. Our segmentation procedure for MRIs from participants ranging from 2 weeks to 89 years collected from different sites was validated in previous studies.^28^^,^^29^^,^^49^ The segmented regions were assembled into a single MRI volume we will refer to as the “segmented head MRI volume.” Figure 1(a) shows a 3D rendering of the T1W volume from a 3-month-old infant with a cutout revealing the segmented MRI volume. The realistic head model represents the geometry of the head and allows us to differentiate optical properties of different tissue types.

### 2.4 Mesh Generation

A finite element tetrahedral mesh was constructed from the segmented head MRI volume. Figures 1(b) and 1(c) displayed meshes that were produced using the iso2mesh toolbox with CGAL 3.6 mesh program (v2m function).^58^ Tetrahedral meshes accurately represent the boundaries of complex three-dimensional volumetric tissues and increase the accuracy in modeling photon propagation in complex mediums such as the head and brain.^59^ The finite element volumetric meshes have nodes that represent the voxel locations for the tetrahedra, a four-element matrix representing the corners of each tetrahedron, and a vector representing the media type from the segmented head MRI volume. A mesh was generated for each segmented head MRI volume. Figure 1(b) shows an example of the dense meshes. The number of nodes, elements, and tetra volume was calculated for the infants (2 weeks to 2 years), children (4 and 12 years), and adults (Fig. S1 in the Supplemental Material). We will refer to this as the “segmented finite element mesh.” We used the mesh to locate points on the scalp that were closest to the electrode positions and for the segmented finite element mesh for the MMC computer program.

### 2.5 Scalp Locations

#### 2.5.1 Virtual electrodes placement

**Fig. 2:**
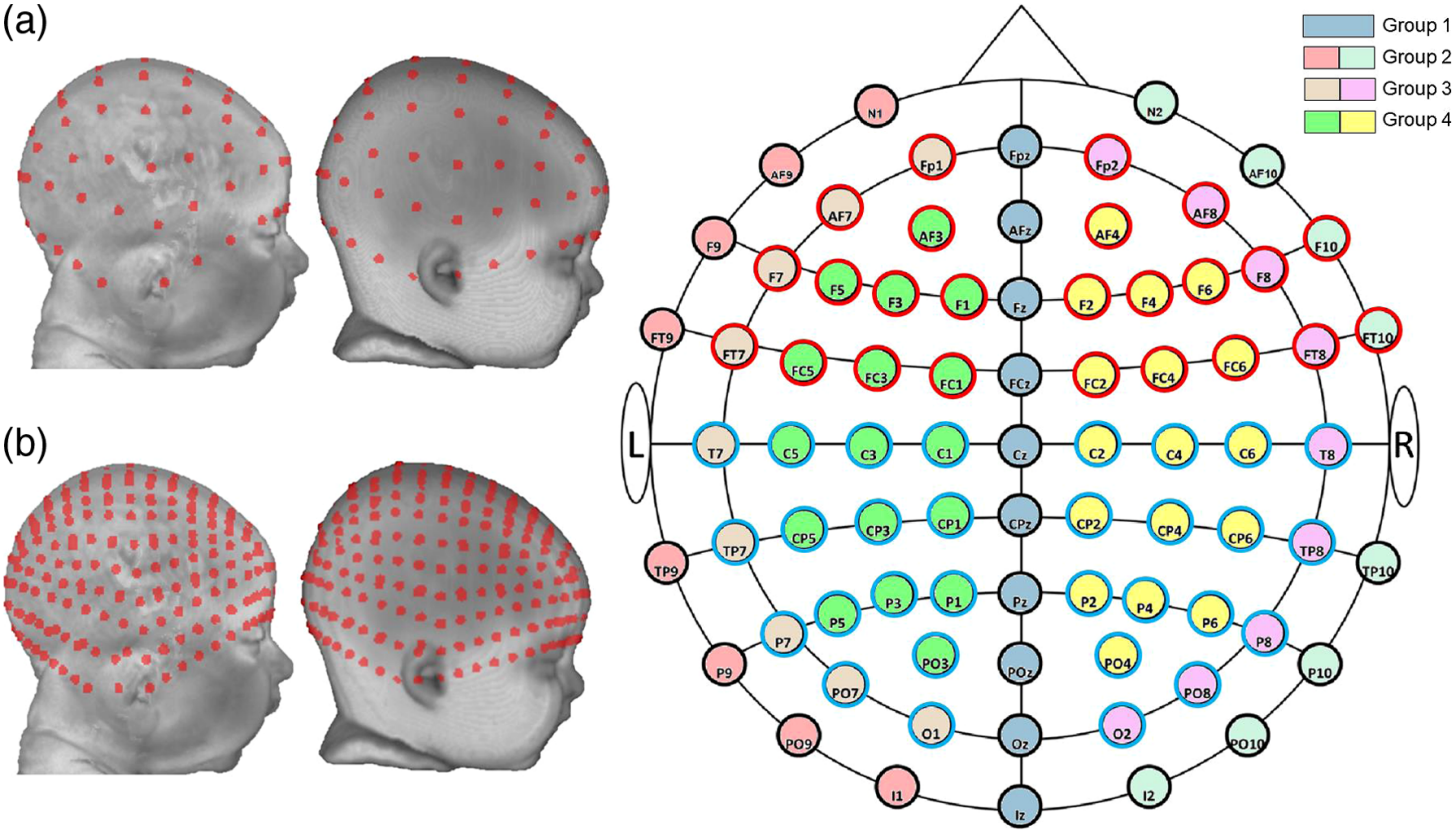
Virtual electrode placement. (a) 10–10 virtual electrode placement. From left to right: a 3-month individual head model, an average template for 3-month-olds, and a 2D layout of the 10–10 system. 10–10 electrodes were divided into four groups based on circumference location and expected similarity of scalp-to-cortex distances. The electrodes were further divided based on the hemisphere in the analysis that examined hemispheric differences in scalp-to-cortex distances. The groups were color-coded (fill colors) in circumference-location-by-hemisphere divisions. Electrodes were divided into frontal electrodes (F electrodes; red outline) and central/posterior (C, T, P, and O electrodes; blue outline) to examine the anterior-to-posterior differences in scalp-to-cortex distances. See text for an explanation of the electrode groupings. (b) 10–5 virtual electrode placement. From left to right: the same 3-month individual head model and the 3-month average template.

The locations for the 10–10 and 10–5 electrode systems were constructed on each head MRI volume. Figure 2 shows the placements of 10–10 and 10–5 electrode positions. First, we manually marked cranial fiducial points using MRIcron:^56^^,^^57^ nasion (Nz), Vz, inion (Iz), left preauricular point (LPA), right preauricular point (RPA), left mastoid, and right mastoid. The fiducial definitions were defined by procedures described in Refs. 11 and 60. The manual marking was done by one person (e.g., research assistant or one of the authors), and the marks were visually inspected by one of the authors for accuracy and corrected if necessary. The coordinates of the fiducials were transferred onto the scalp mesh. Next, we calculated 81 virtual electrode positions based on the unambiguously illustrated 10–10 system.^11^ Details for constructing the 10–10 locations are described in Ref. 60 and the Supplemental Material. The electrode positions were visually inspected by one of the authors. We simulated a total of 358 electrodes on 10–5 locations by calculating center points between 10–10 positions. The electrode positions also were computed for the average MRI templates.

The individual electrode locations were divided into groups for analyses based on anatomical landmarks.^11^ There were divided into four groups based on sagittal curves, with groups of electrodes that we expected to have similar scalp-to-cortex distances. Group 1: electrodes on the central curve (Nz-Cz-Iz). They are over the interhemispheric fissure. Group 2: electrodes on the left and right curves between Nz and Iz (left: N1-LPA/T9-I1; right: N2-RPA/T10-I2). Group 3: electrodes on the left and right curves between Fpz and Oz (left: Fp1-T7-O1; right: Fp2-T8-O2). Group 4: the remaining electrodes enclosed by the central curve and the left and right curves between Fpz and Oz. We expected the first two electrode groups to have greater distances based on their lowest position on the scalp; group 3 to have intermediate distances; group 4 to have the smallest distances. The electrode groups were further divided based on the hemisphere in the analysis that explored hemispheric differences in distances. Electrode locations in groups 3 and 4 could also be divided by the coronal curve (T7-T8). We group these electrodes into frontal electrodes (F electrodes) and central/posterior electrodes (C, T, P, and O electrodes) for the analysis that examined the frontal-to-occipital differences in distances. The electrode groups are displayed in Fig. 2.

#### 2.5.2 Source–detector channels

Source–detector channel locations were defined using the electrode combinations centered on each 10–10 electrode. The 10–10 electrode locations were centered between surrounding adjacent pairs of 10–10 or 10–5 electrode locations. There were 251 source–detector pairs formed with adjacent 10–10 electrodes, ${\text{Mean}}_{\text{Separation}}=58.0\;\mathrm{mm}$; SD=14.2, and 251 pairs formed with adjacent 10–5 electrodes, ${\text{Mean}}_{\text{Separation}}=28.9\;\mathrm{mm}$; SD=7.1. The channel locations were used to estimate the “S-D channel DOT fluence” described below.

### 2.6 Cortical Locations

Three stereotaxic atlases were constructed for each individual MRI. The atlases delineate cortical lobes or more specified locations within the lobes. We created a 1.5-cm spherical mask around each electrode or channel location to standardize the region to be identified in the atlases. The atlases and spherical masks were used with the scalp projection method (described below) to describe the correspondence between the scalp electrode positions and corresponding cortical structures in the individual’s own brain space with various spatial resolutions.^25^ They were also used with the “S-D channel DOT” estimates (described below) to identify the anatomical structures being sampled by the fluence distribution.

#### 2.6.1 Stereotaxic atlases

Three atlases were constructed for each individual MRI to delineate anatomical regions that can be used to identify brain locations corresponding to scalp electrode positions or DOT sensitivity patterns. Details of the atlas constructions may be found elsewhere.^25^^,^^30^^,^^61^ The first atlas was the LONI Probabilistic Brain Atlas (LPBA40),^62^ which contains 56 areas from the cortical and subcortical regions, brainstem, and cerebellum. The second was the Hammers atlas, based on MRIs from the IXI MRI database,^63^ which consists of 83 areas defined from the cortex, subcortical, brainstem, and cerebellum.^64^ We used a majority vote fusion procedure that combines labeled segments from manually segmented MRIs to produce atlases that identify an anatomical area for each brain voxel of the individual MRI. The third atlas was an automatically constructed lobar atlas that identifies the major cortical lobes (e.g., frontal), some sublobar cortical (e.g., fusiform gyrus), subcortical (e.g., striatum), cerebellum, and brainstem. The atlas was constructed by extracting and combining common areas from the LPBA40 and Hammers atlases constructed in the average template space. The individual MRI was linearly registered to the age-appropriate average template, and the age-appropriate lobar atlas was transformed using the linear registration matrix into the individual MRI space. Linear registration was performed using the FSL FLIRT function.^65^ The approach has produced good correspondence between the manually segmented average template atlas and the transformed atlas.^25^^,^^30^^,^^61^

#### 2.6.2 Spherical masks

**Fig. 3:**
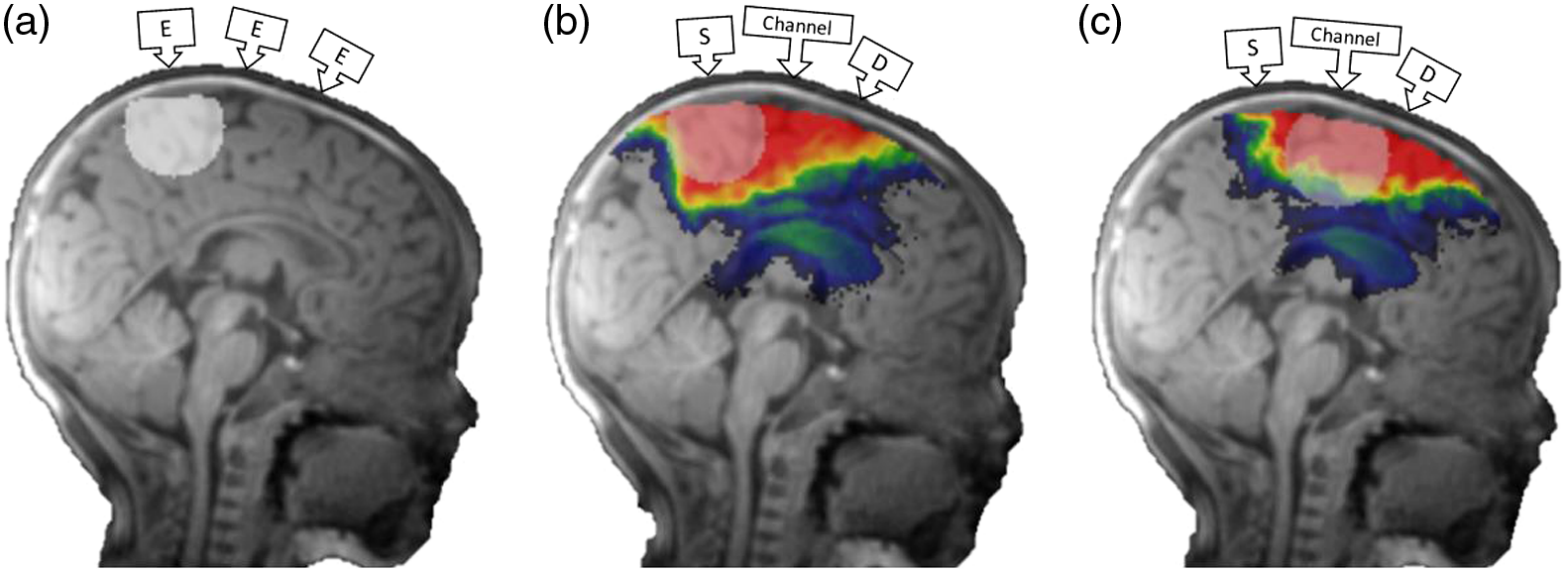
Methods for determining scalp-location-to-ROI mapping. The MRI was taken from a 3-month infant. (a) An example of a 1.5-cm-radius spherical mask created around an electrode location for the spatial scalp projection procedure. (b) An illustration of a spherical mask created around a source location for the direct DOT procedure. (c) An illustration of a spherical mask created around a channel location for the S-D channel DOT procedure. Monte Carlo photon migration simulations were used to estimate fluence distributions for individual 10–5 electrode locations (direct DOT) and 10–10 channel locations (S-D channel DOT). The red area represents greater fluence.

We created a sphere with a 1.5-cm radius around each 10–10 electrode and channel location. The decision of the sphere size was based on previous studies.^32^^,^^33^ It is hypothesized that the DOT fluence distribution reaches about 3 cm deep into the brain with a 2-cm source–detector channel separation. Figure 3 shows examples of the spherical volumes that were used as masks for determining the correspondence between the scalp and brain locations. We identified the atlas ROIs that intersected with the masks and computed the voxel numbers in the sphere of these ROIs. Hence, there was a distribution of atlas ROIs with various voxel numbers that were mapped to each electrode and channel location.

### 2.7 DOT Sensitivity Analyses

The DOT fluence distribution was estimated from Monte Carlo simulations using the Monte Carlo eXtreme package MCX.^66^^,^^67^ Details of the simulations are presented in Ref. 37 and the Supplemental Material. The output from the Monte Carlo simulation contained the fluence across the entire MRI volume separately for each optode. Figure 3(b) shows the direct DOT fluence plotted on an individual subject MRI for a single source-optode location. We computed S-D channel DOT fluence by multiplying the source-optode fluence distribution by the detector-optode fluence distribution. This represents the DOT fluence sensitivity for a photon channel from the source to the detector (PMDF;^10^ or 3-point Green’s function).^35^^,^^36^ Figure 3(c) shows the S-D channel DOT fluence plotted on an MRI for a single source–detector pair.

### 2.8 Spatial Scalp Projection

We performed scalp projections from each 10–10 or 10–5 electrode position on the scalp to the brain surface. These projections were used to measure the distance between each scalp electrode and the brain surface and to examine electrode scalp location to anatomical ROI correlation.^19^^,^^25^^,^^31^^–^^33^ The brain locations underlying each electrode were determined by an algorithm in which a spatial projection from the electrode scalp surface to the brain center was done. The point where the projection intersected the cortex was identified. These projections were done with the scalp and brain elements of the segmented finite element mesh and mesh manipulation tools from the iso2mesh toolbox.^58^

### 2.9 Scalp-to-Cortex Distance

We computed the distance from scalp electrode or channel locations to cortical surface for each individual from all groups. Figure 3(a) shows a 1.5-cm radius spherical mask that was created at the point where the scalp projection intersected the brain. The scalp projection distance was the distance between the scalp electrode position and the closest intersection point on the brain surface. The direct DOT distance was based on the direct DOT fluence. Figure 3(b) shows the spherical mask overlaid on the direct DOT fluence distribution at a source–electrode (optode) location. The maximum direct DOT fluence in the spherical mask was identified. The direct DOT distance was the distance between the scalp electrode position and the location of the brain voxel with the maximum direct DOT fluence in the spherical mask. The S-D channel DOT distance was based on the S-D channel DOT fluence. Figure 3(c) shows the spherical mask overlaid on the S-D channel DOT fluence distribution at a source–detector channel location. The S-D channel DOT distance was the distance between the scalp channel position and the location of the voxel with the maximum S-D channel DOT fluence in the spherical mask.

Data analyses examined the effect of age, estimation method, and electrode location on the scalp-to-cortex distance. A three-way ANOVA was conducted to examine the main effect of the three factors and their interaction effects. This was followed by additional analyses assessing the age-related differences in the distance measure across estimation methods, and the electrode-location differences in distances across estimation methods in infants, children, and adults. *Post-hoc* analyses were also performed to explore hemispheric asymmetry in scalp projection and S-D channel DOT distances across electrode positions and age groups.

### 2.10 Scalp-Location-to-ROI Mapping

The scalp projection, direct DOT, and the S-D channel DOT sensitivity were used to generate a look-up procedure that links the scalp electrode or channel locations to the lobar, Hammer, and LPBA40 atlas ROIs. A 1.5-cm mask was placed on the scalp projection to the brain intersection, maximum point of the direct DOT fluence, or maximum point of the S-D channel DOT fluence. Figure 3(a) shows a sphere surrounding the scalp projection to the brain intersection; Fig. 3(b) shows a sphere surrounding the source-optode to the maximum direct DOT fluence location; Fig. 3(c) shows a sphere surrounding the channel location to the S-D channel DOT fluence. The anatomical ROI(s) of each voxel in the respective spheres were recorded. The percentage of voxels in each ROI that intersected with the spherical masks was computed. We created tables for each age that listed the ROIs and the percentage of voxels in each ROI that intersected with the spherical masks created around the 10–10 electrode (scalp projection) and channel locations (S-D channel DOT). The scalp projection look-up table details the spatial correspondence between the scalp electrode location and anatomical brain regions. The S-D channel DOT look-up table illustrates the S-D channel DOT sensitivity to cortical regions. These tables serve as references for NIRS or fNIRS users to make age-specific decisions of optode placement or evaluate channel-to-ROI mapping for existing channel configurations.

### 2.11 Additional Measures and Analyses

Several methods and results are presented in the Supplemental Material. These include tMCimg^5^ simulations in all individual MRIs and age-appropriate average templates and MMC simulations^59^^,^^66^^,^^68^ in 3-month- and 6-month-old individual MRIs and average templates. We compared the scalp-to-cortex distance estimations obtained from the MCX, tMCimg, and MMC simulation packages. fNIRS optodes’ location decider (fOLD) channels: We used a set of electrode pairs to define source–detector channels from the 130 channel locations specified in the fOLD.^42^ We computed S-D channel DOT fluence in selected age groups (3 months, 6 months, and 20 to 24 years) for the 130 channel locations using fluence estimated from MCX simulations.^42^ We provided a look-up table that presented the specificity of each fOLD channel to the underlying ROI(s). We also compared scalp-to-cortex distances among simulation methods and brain model types.

## 3 Results

### 3.1 Scalp-to-Cortex Distance

**Fig. 4:**
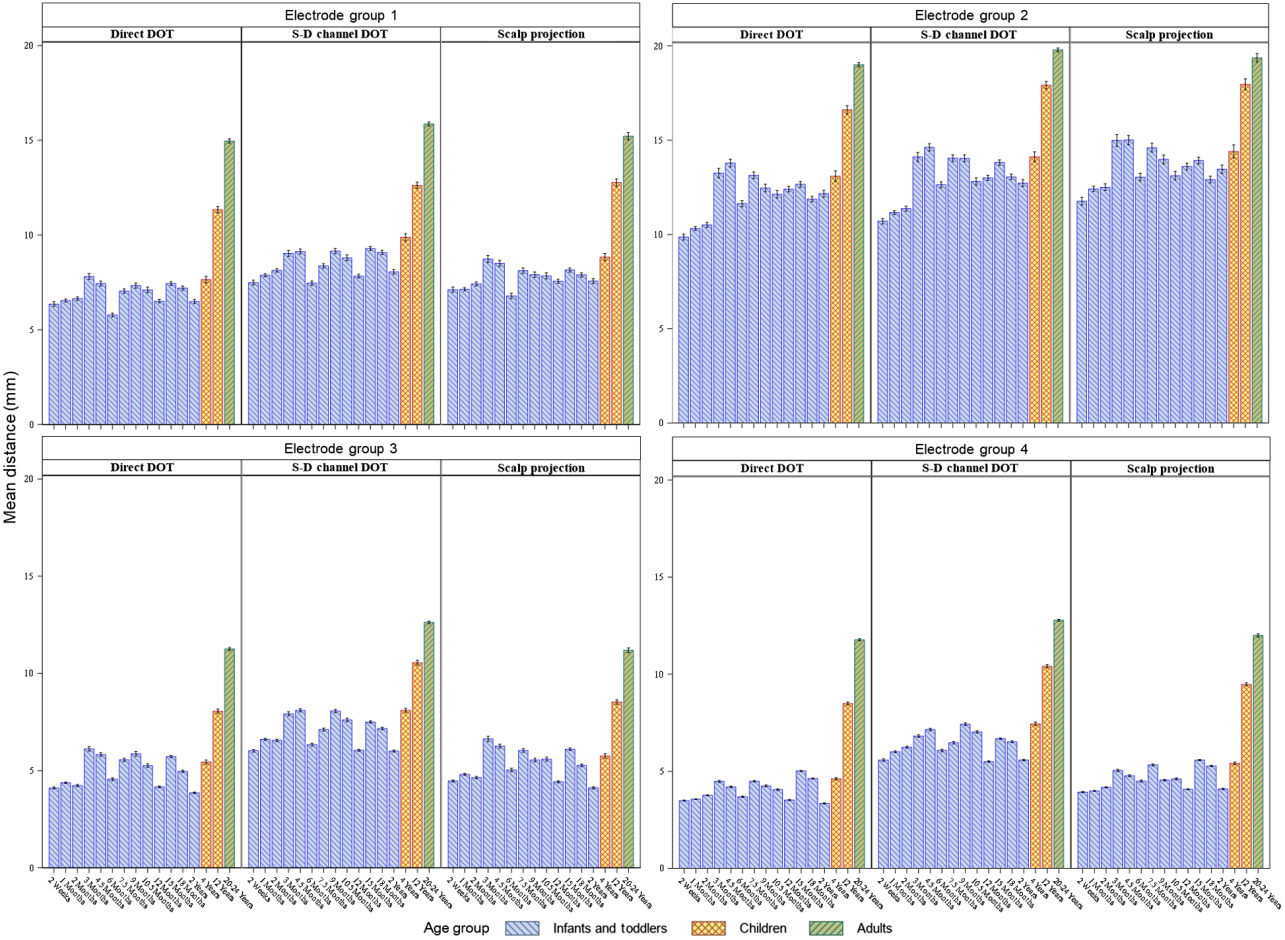
Mean scalp-to-cortex distances averaged across individual electrode locations by age group, estimation method, and electrode group. The distances were estimated using individual MRIs.

The scalp-to-cortex distances were analyzed as a function of age group, estimation method, and electrode group. Parameter estimates from all ANOVA models were displayed in Table S2 in the Supplemental Material. Figure 4 shows the average scalp-to-cortex distances by age groups, estimation methods, and electrode groups. A three-way ANOVA (model 1) was performed to examine the effect of age group (2 weeks to 20 to 24 years), electrode group (four groups), estimation method (scalp projection, direct DOT, S-D channel DOT), and their interaction effects. There was a significant main effect of age group, F(15,12,144)=1652.55, p<0.001, electrode group, F(6,12,144)=14,821.90, p<0.001, and estimation method, F(2,12,144)=1150.78, p<0.001. There was a significant interaction effect of age-by-electrode-group, F(45,12,144)=18.34, p<0.001, age-by-method, F(30,12,144)=4.65, p<0.001, method-by-electrode-group, F(6,12,144)=107.26, p<0.001, and age-by-electrode-group-by-method three-way interaction, F(90,12,144)=1.59, p<0.001. The mean distance averaged across estimation methods and electrode locations was greatest in the adults, followed by 12-year-olds, p values <0.001. The mean distance at age 4 years was greater than the distance at most of the infant and toddler ages, p values <0.05, except for 3-month- and 4.5-month-olds. The youngest age group (2 weeks) had the smallest distance, p values <0.001. However, the mean distance did not show a systematic increase with age during infancy. The mean distance across age groups and estimation methods was furthest from the cortex at the left- and right-lowest circumference electrode positions (group 2), followed by the midline electrodes (group 1), the second-lowest circumference electrodes (group 3), and the remaining electrodes (group 4), p values <0.001. The mean distance averaged across age groups and electrode locations was largest for the S-D channel DOT estimation method, followed by scalp projection, and direct DOT in descending order, p values <0.001. Figure 4 shows that the age differences among infant and toddler groups (2 weeks to 2 years) differed by estimation methods and electrode locations. Figure S3 in the Supplemental Material provides an additional visualization of distances across individual electrode positions estimated using the three methods. We conducted two sets of follow-up analyses (models 2a, 2b, 3a, 3b) to further examine the age-by-electrode-location interaction effect on the distances estimated using scalp projection and S-D channel DOT, as the two methods have direct applications to NIRS measurements.

**Fig. 5:**
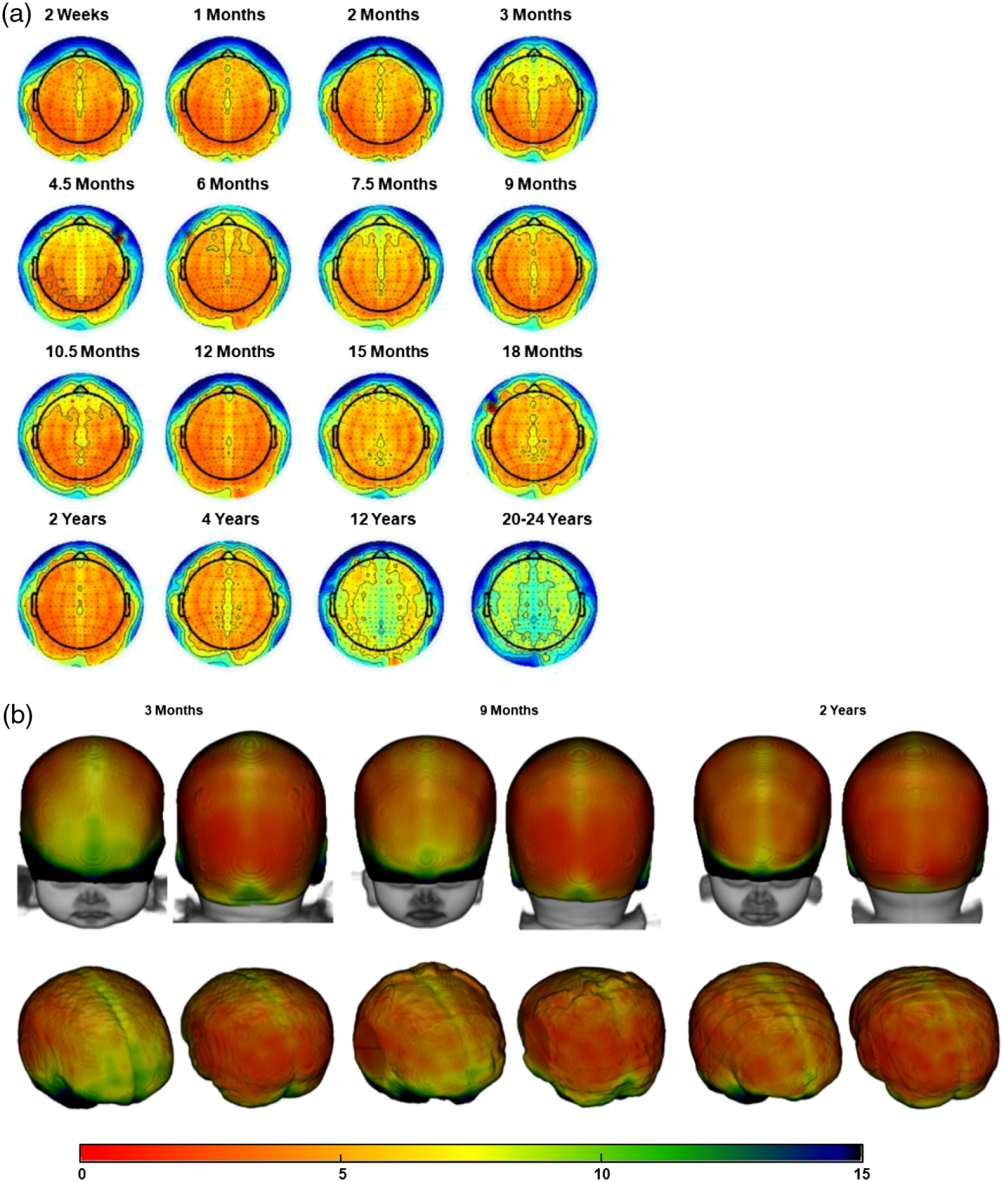
Mean distance from the scalp to the cortical surface by electrode locations estimated using scalp projection. For the visualization purpose, estimations for the 10–5 electrode locations were displayed. The color bar denotes the distance range for all figure types. (a) Scalp topographical maps for all age groups. Darker red represents closer scalp-to-cortex distances, and darker blue indicates greater distances. (b) 3D rendering of mean distances on the heads (top row) and on the brains (bottom row) at 3 months, 9 months, and 2 years, selected as examples. Head and brain models were generated using age-matched average templates.

**Fig. 6:**
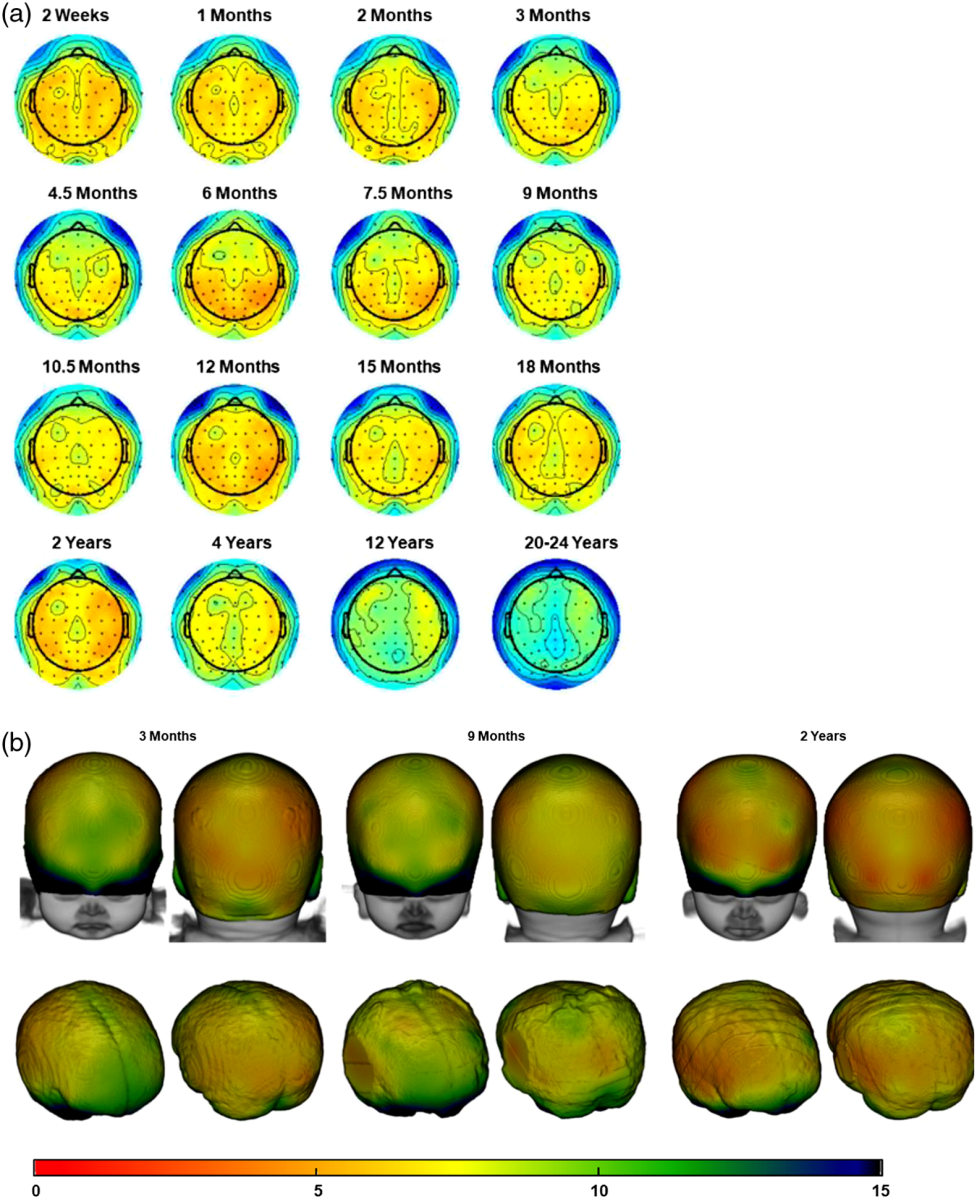
Mean S-D channel DOT distances at 10–10 channel positions. The color bar denotes the distance range for all figure types. (a) Scalp topographical maps for all age groups. Darker red represents closer scalp-to-cortex distances, and darker blue indicates greater distances. (b) 3D rendering of mean distance on the heads (top row) and the brains (bottom row) at 3 months, 9 months, and 12 years, selected as examples. Head and brain models were generated using age-matched average templates.

We further examined how scalp-to-cortex distances varied across scalp positions and age groups. Figure 5 shows two-dimensional (2D) scalp topographical maps plotted with the scalp projection distance represented by the color map separately by age, and 3D renderings of the distance on the head for three selected ages. Figure 5 confirmed the main effect of age group and electrode group found in the previous analysis. The figure further revealed variations in distance between anterior/frontal positions and posterior positions. The shift from the frontal-midline electrodes to the other scalp positions was evident in several groups. Figure 6 shows 2D maps and 3D renderings for the S-D channel DOT scalp-to-cortex distance. The distance of the maximum S-D channel DOT fluence was larger than the scalp projections distance at all ages as shown in the previous analyses (c.f. Fig. 4). The anterior-to-posterior variation was also found with the S-D channel DOT distance for most of the age groups.

We conducted two-way ANOVAs to depict the age-by-electrode-location interaction effects. As described in Sec. 2.5.1, we regrouped the electrodes in groups 3 and 4 with the purpose of exploring the anterior-to-posterior variation in distance. The first ANOVA (model 2a) tested the effect of individual age groups and electrode groups on scalp projection distances. There was a significant effect of age groups, F(15,3816)=374.64, p<0.001, electrode group F(3,3816)=6393.45, p<0.001, and age-by-electrode-group interaction effect, F(45,3816)=7.54, p<0.001. The scalp-to-cortex distances of the group two (lowest-circumference) electrodes were the largest, followed by group one (midline) electrodes for all age groups, p values <0.001. The group three electrodes at frontal locations were more distant to the cortex than group four electrodes at central, temporal, parietal, and occipital locations for all infant age groups, p values <0.001. The difference between groups 2 and 3 electrode locations was not significant for 4 years, 12 years, and 20 to 24 years. The second ANOVA (model 2b) tested the effect of age groups and electrode groups on S-D channel DOT distances. There was a significant effect of age groups, F(15,4164)=690.39, p<0.001, electrode group F(3,4164)=3517.76, p<0.001, and age-by-electrode-group interaction effect, F(45,4164)=12.69, p<0.001. The distances at group 2 (lowest-circumference) electrodes were the largest for all age groups, p values <0.001. Group 1 (midline) electrodes were more distant to the cortex than both group 3 (frontal) and group 4 (central, temporal, parietal, and occipital) electrodes for most of the age groups, p values <0.05, but the distances between group 1 and group 3 electrodes were not significantly different for 3 months, 4.5 months, 6 months, and 7.5 months. Group 3 electrodes had a greater distance than group 4 electrodes, p values <0.05, except for 2 weeks, 1 month, 2 months, 18 months, 2 years, and 4 years. The findings indicate that the differences in scalp-to-cortex distances between anterior/midline and posterior electrode positions varied by age and estimation methods. There was an anterior/midline-to-posterior decrease in scalp-to-cortex in most of the infant groups.

**Fig. 7:**
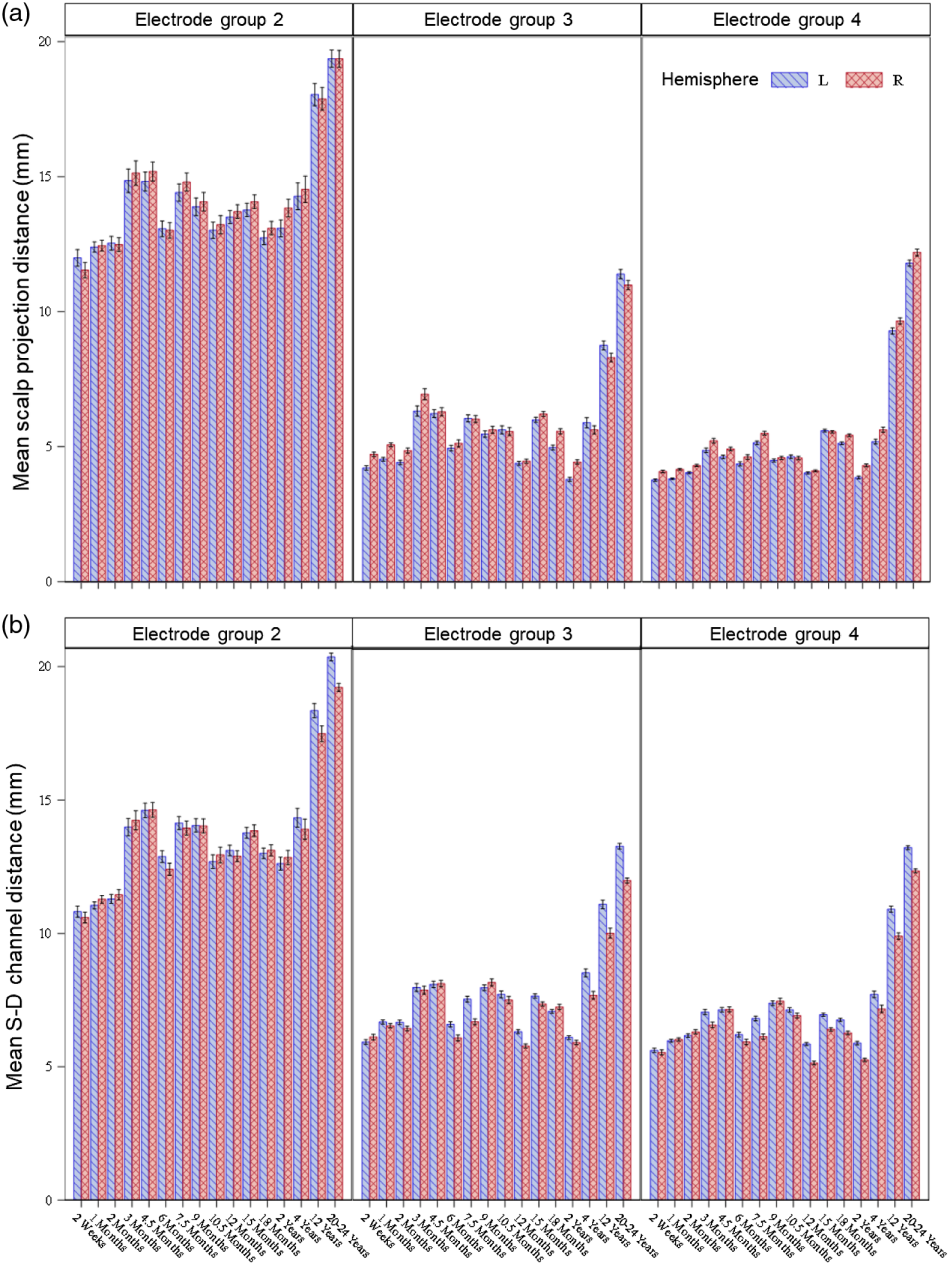
Mean scalp-to-cortex distances across individual 10–10 electrode/channel locations by circumference locations, and hemispheres for individual age groups. Electrode locations at group one (c.f. Fig. 2) were excluded from the analyses. (a) Mean scalp projection distance across individual electrode locations by circumference locations and hemispheres for individual age groups. (b) Mean S-D channel DOT distance across individual channel locations by circumference locations and hemispheres for individual age groups.

We investigated whether scalp-to-cortex distances varied by circumference location and hemisphere across age groups. The analyses excluded electrodes in the midline positions (group 1). Figure 7 displays mean distances by age group, hemisphere, and electrode group for the scalp projection distance [Fig. 7(a)] and S-D channel DOT estimations [Fig. 7(b)]. The first three-way ANOVA (model 3a) tested the effect of age groups, electrode group, and hemisphere (left and right) on the scalp projection distances. There was a significant effect of age group, F(15,5724)=432.03, p<0.001, electrode group, F(2,5724)=15,661.50, p<0.001, and age-by-electrode-group interaction, F(30,5724)=11.39, p<0.001. These findings are consistent with the previous results. There was a significant effect of hemisphere, F(1,5724)=19.35, p<0.001. The mean distance in the right hemisphere was greater than the left hemisphere, p<0.001. The age-by-hemisphere interaction effect, electrode-group-by-hemisphere effect, nor the three-way interaction effect was significant. The second three-way ANOVA (model 3b) examined the effect of age group, electrode group, and hemisphere on the S-D channel DOT distances. There was a significant effect of age group, F(15,6246)=870.31, p<0.001, electrode group, F(2,6246)=9248.99, p<0.001, and age-by-electrode-group interaction, F(30,6246)=14.87, p<0.001. In addition, there was a significant effect of hemisphere, F(1,6246)=44.49, p<0.001, age-by-hemisphere interaction, F(15,6246)=6.20, p<0.001, and electrode-group-by-hemisphere interaction effect, F(2,6246)=3.14, p<0.04. The age-by-electrode-group-hemisphere interaction effect was not significant. Across all electrode groups, the mean distance was greater in the left than right hemisphere for 6 months, 7.5 months, 12 months, 4 years, 12 years, and 20 to 24 years, p values <0.05, whereas the hemispheric difference was not significant for the rest of the age groups. Across all age groups, the distance was greater in the left than right hemisphere for group 3 (second-lowest circumference) and group 4 (remaining) electrodes, p values <0.001, but not for group 2 (lowest circumference) electrodes.

### 3.2 Additional Analyses on Scalp-to-Cortex Distance

The Supplemental Material provides additional results on the effect of sex, head model type, and additional estimation methods on age-related differences in scalp-to-cortex distance. Figure S4 in the Supplemental Material displays the effects of sex, age group, and electrode group on S-D channel DOT distances estimated using individual head models. It shows that the S-D channel DOT distances were greater in males than females in some age groups but not others. Figure S5 in the Supplemental Material shows that the S-D channel DOT distances by age group and electrode group computed from age-matched average templates (Fig. S5A in the Supplemental Material) and individual head models (Fig. S5B in the Supplemental Material). The distances for the participant-based averages (cf. Fig. 4) were similar to the distances calculated from the average MRI template for the same age. Figures S6 and S7 in the Supplemental Material show comparisons among scalp-to-cortex distances estimated using MCX, tMCimg, and MMC simulation methods with 10–10 channel locations, as well as MCX simulation with fOLD channel locations. They showed that MCX, MMC, and tMCimg estimation methods produced comparable S-D channel DOT distances for individual MRIs and average templates at 3 months and 6 months. The distances estimated using MCX fOLD with average templates were larger than distances calculated from other methods with individual head models and average templates at the example age groups.

### 3.3 Scalp-Location-to-ROI Mapping

The scalp projection and the S-D channel DOT sensitivity were used to generate look-up tables that show the cortical ROIs for each of the 10–10 electrodes. A supplemental Excel spreadsheet is presented with this information (see Supplemental Table S3). This table contains each estimation type and age combination (e.g., spatial projection 2-0 weeks, spatial projection 1-0 month, source–detector DOT 2-0 weeks, source–detector DOT 1-month, etc.). Each table has one row for each electrode containing columns for the electrode name and the cortical ROIs for the lobar, Hammers, and LPBA40 atlases. These tables could be used to find a specific scalp-location-to-ROI combination for each of the ages in the study. The scalp projection and S-D channel DOT estimations yielded overlapping and considerably different scalp-location-to-ROI mappings. For example, between-method discrepancy was found at 32 scalp locations where the electrode was mapped to at least one different ROI for the 3-month group. The discrepancy was found at 29 scalp locations for the 20- to 24-year-olds. NIRS/fNIRS users are recommended to refer to the S-D channel DOT look-up table, as the channel-to-ROI correspondence was estimated based on the sensitivity of the channel location to the brain region.

**Fig. 8:**
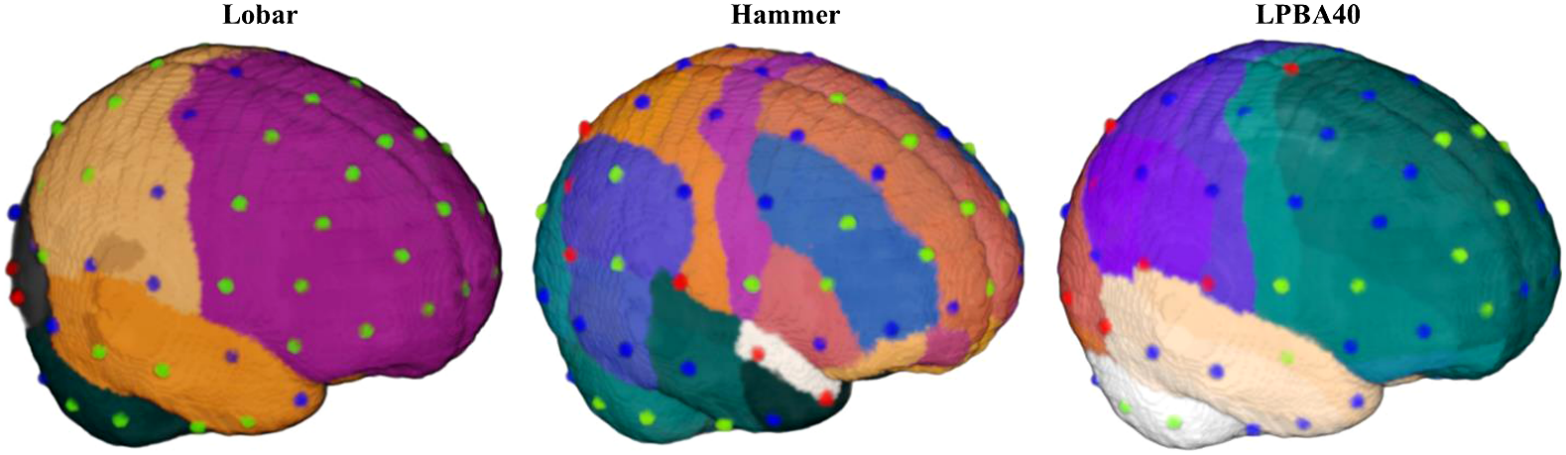
Age-group differences in the correspondence between scalp channel location and the underlying brain ROI from the lobar, Hammer, and LPBA40 atlas. Scalp 10–10 channel locations are overlaid on the average template for age 2 years. Channel locations colored in green were mapped to the same ROI(s) across all age groups. Blue indicates that the channel location was mapped to at least one ROI across age groups, and red shows that different ROIs were corresponded to the channel location across age groups. The information on between-age consistency was based on Table 2.

The channel-location-to-ROI correspondences showed stability over age for some of the channel locations based on the S-D channel DOT estimation. Table 2 and Fig. 8 summarize the differences in channel-to-ROI mapping across age groups. For each channel location, the table listed ROIs that mapped to the location with sufficient confidence (defined as having at least 25% of brain voxels intersected with the 1.5-cm-radius sphere created around the channel location) and the age groups with the ROI that survived the threshold. The channel locations were color-coded to show different levels of between-age consistency in Fig. 8. The lobar atlas has macrostructural segmentations which resulted in similar channel-to-ROI correspondence across ages. Most of the channel locations (52 out of 81 channels) were mapped to one lobar atlas ROI for all age groups. Some channels were sensitive to more than one lobar ROIs in younger age groups but were mapped to one lobar ROI in older age groups (CZ, PZ, OZ, F9, F10, T10, FT7, FT8, O1, O2, C5, C6, C1, C2, CP5, and CP6). For example, O1 (left) and O2 (right) were mapped to the cerebellum, fusiform gyrus, occipital lobe, and parietal lobe for the infant groups but only to the occipital lobe for 20- to 24-year-olds. Some channels were sensitive to more than one lobar ROIs only in older age groups (POZ, PO9, I1, and I2). For example, I2 corresponded to both the right cerebellum and occipital lobe in 12- and 20- to 24-year-olds but only to the cerebellum in younger ages. Other channels had more discrepant channel-to-ROI mappings over age (IZ, P7, P8, PO7, PO8, C3, C4, PO3, and PO4).

**Table 2:** Atlas locations corresponding to the scalp channel locations estimated using S-D channel DOT method. For each channel location, we listed atlas ROI that had at least 25% of its voxels intersected with the 1.5-cm-radius sphere created around the channel location (c.f. Supplemental Table S3). We also listed the age groups with the ROI that survived the threshold.

Electrode number	Electrode name	Lobar atlas ROI	Ages	Hammer atlas ROI	Ages	LPBA40 atlas ROI	Ages
1	NZ	Frontal	All	Medial orbital gyrus	All	Superior frontal gyrus	All
				Superior frontal gyrus	2 weeks to 4.5 months, 20 to 24 years		
2	FPZ	Frontal	All	Superior frontal gyrus	All	Middle frontal gyrus	2 months
						Superior frontal gyrus	All
3	AFZ	Frontal	All	Superior frontal gyrus	All	Superior frontal gyrus	All
4	FZ	Frontal	All	Superior frontal gyrus	All	Superior frontal gyrus	All
5	FCZ	Frontal	All	Superior frontal gyrus	All	Superior frontal gyrus	All
6	CZ	Frontal	All	Postcentral gyrus	1m	Precentral gyrus	2 weeks to 4.5 months; 7.5 months to 20 to 24 years
		Parietal	2 weeks to 2 months	Precentral gyrus	All	Superior frontal gyrus	2 months to 20 to 24 years
				Superior frontal gyrus	3 to 12 months; 18 months to 20 to 24 years	—	—
				Superior parietal gyrus	2 weeks		
7	CPZ	Parietal	All	Postcentral gyrus	6 months; 12 years, 20 to 24 years	Postcentral gyrus	3 months to 20 to 24 years
				Superior parietal gyrus	All	Precuneus	2 weeks to 3 months; 15 months
						Superior parietal gyrus	All
8	PZ	Occipital	2 weeks; 1 month	Cuneus	2 weeks; 1 month	Precuneus	2 months to 20 to 24 years
		Parietal	All	Superior parietal gyrus	All	Superior parietal gyrus	1 months to 20 to 24 years
9	POZ	Occipital	All	Cuneus	All	Cuneus	All
		Parietal	20 to 24 years	Lateral remainder of occipital lobe	2 weeks to 2 months; 7.5, 12 months	Superior occipital gyrus	2 months to 20 to 24 years
				Superior parietal gyrus	12 years		
10	OZ	Fusiform	2 weeks, 1, 3, 4.5, 9, 10.5, 15, and 18 months	Cuneus	2 months to 20 to 24 years	Cuneus	3 months to 20 to 24 years
		Occipital	All	Lateral remainder of occipital lobe	All	Lingual gyrus	1, 3, and 4.5 months
				Lingual gyrus	2 weeks, 1, 3, and 4.5 months	Middle occipital gyrus	2 months, 4.5 months to 4 years, 20 to 24 years
11	IZ	Cerebellum	2 weeks to 4 years	Cerebellum	2 weeks to 12 years	Cerebellum	2 weeks to 12 years
		Fusiform	10.5 months, 2 y, 4, 20 to 24 years	Lateral remainder of occipital lobe	12, 20 to 24 years	Lingual gyrus	12, 20 to 24 years
		Occipital	12, 20 to 24 years	Lingual gyrus	12, 20 to 24 years		
12	N1	Frontal	All	Anterior orbital gyrus	All	Middle frontal gyrus	2 weeks to 2 months
				Medial orbital gyrus	All	Middle orbitofrontal gyrus	4.5 months to 20 to 24 years
				Superior frontal gyrus	2 weeks; 1 month	Superior frontal gyrus	All
13	AF9	Frontal	All	Anterior orbital gyrus	2 weeks to 9 months; 12 months, 2 years, 4 years	Lateral orbitofrontal gyrus	4.5, 10.5, 15 months; 2 years to 20 to 24 years
				Lateral orbital gyrus	4.5, 10.5, 15 months; 2 years to 20 to 24 years	Middle orbitofrontal gyrus	3 to 12 months; 18 months to 4 years
				Medial orbital gyrus	12 months		
14	F9	Frontal	2 weeks to 3 months	Anterior temporal lobe, lateral part	4.5 months to 20 to 24 years	Middle temporal gyrus	10.5, 15 months; 20 to 24 years
		Temporal	All	Anterior temporal lobe, medial part	12 and 18 months, 2 and 4 years	Superior temporal gyrus	All
				Superior temporal gyrus, anterior part	9, 10.5, 15 months, 12 years, 20 to 24 years		
15	FT9	Temporal	All	Anterior temporal lobe, lateral part	3 months to 20 to 24 years	Inferior temporal gyrus	All
				Medial and inferior temporal gyri	All	Middle temporal gyrus	1 month to 20 to 24 years
						Superior temporal gyrus	2 weeks to 3 months
16	T9	Temporal	All	Medial and inferior temporal gyri	All	Inferior temporal gyrus	All
						Middle temporal gyrus	2 weeks to 3 months; 20 to 24 years
17	TP9	Cerebellum	All	Cerebellum	All	Cerebellum	All
18	P9	Cerebellum	All	Cerebellum	All	Cerebellum	All
19	PO9	Cerebellum	All	Cerebellum	All	Cerebellum	All
		Parietal	12 years; 20 to 24 years	Lateral remainder of occipital lobe	12 years; 20 to 24 years	Inferior occipital gyrus	12 years; 20 to 24 years
20	I1	Cerebellum	All	Cerebellum	All	Cerebellum	All
		Occipital	12 years; 20 to 24 years	Lateral remainder of occipital lobe	6 months; 12 years; 20 to 24 years	Inferior occipital gyrus	6 and 10.5 months; 12 years; 20 to 24 years
		Parietal	12 years				
21	N2	Frontal	All	Anterior orbital gyrus	3, 6, 7.5 months; 12 years; 20 to 24 years	Middle orbitofrontal gyrus	6, 7.5 months; 2 years to 20 to 24 years
				Medial orbital gyrus	All	Superior frontal gyrus	All
				Superior frontal gyrus	2 weeks to 2 months		
22	AF10	Frontal	All	Anterior orbital gyrus	All	Lateral orbitofrontal gyrus	12 years; 20 to 24 years
				Lateral orbital gyrus	12 years; 20 to 24 years	Middle frontal gyrus	2 weeks, 1 month
				Medial orbital gyrus	7.5 to 12 months; 18 months; 2 years	Middle orbitofrontal gyrus	1 month to 12 years
						Superior frontal gyrus	12 months
23	F10	Frontal	2 weeks to 7.5 months	Anterior temporal lobe, lateral part	9, 10.5, 15 months; 2, 12, 20 to 24 years	Middle temporal gyrus	20 to 24 years
		Temporal	All	Anterior temporal lobe, medial part	4.5 months to 4 years	Superior temporal gyrus	All
				Superior temporal gyrus, anterior part	9, 15, 18 months; 4 years to 20 to 24 years		
24	FT10	Temporal	All	Anterior temporal lobe, lateral part	3 months to 20 to 24 years	Inferior temporal gyrus	All
				Anterior temporal lobe, medial part	1, 2, 7.5, 12 months	Middle temporal gyrus	3 months to 20 to 24 years
				Medial and inferior temporal gyri	6, 9, 10.5, 15 months; 2, 12, 20 to 24 years	Superior temporal gyrus	1 to 3, 6, 7.5 months
25	T10	Cerebellum	10.5 and 15 months	Cerebellum	10.5 and 15 months	Cerebellum	10.5 and 15 months
		Temporal	All	Medial and inferior temporal gyri	All	Inferior temporal gyrus	All
						Middle temporal gyrus	2 weeks to 3 months; 6 months; 20 to 24 years
26	TP10	Cerebellum	All	Cerebellum	All	Cerebellum	All
27	P10	Cerebellum	All	Cerebellum	All	Cerebellum	All
28	PO10	Cerebellum	All	Cerebellum	All	Cerebellum	All
				Lateral remainder of occipital lobe	12, 20 to 24 years	Inferior occipital gyrus	12, 20 to 24 years
29	I2	Cerebellum	All	Cerebellum	All	Cerebellum	All
		Occipital	12, 20 to 24 years	Lateral remainder of occipital lobe	6, 7.5 months; 2 to 20 to 24 years	Inferior occipital gyrus	6, 10.5 months; 2 to 20 to 24 years
						Middle occipital gyrus	20 to 24 years
30	FP1	Frontal	All	Anterior orbital gyrus	2 years	Middle frontal gyrus	All
				Middle frontal gyrus	All		
				Superior frontal gyrus	2 weeks to 2 years; 12 years, 20 to 24 years		
31	AF7	Frontal	All	Lateral orbital gyrus	7.5 months; 2, 4 years	Inferior frontal gyrus	All
				Middle frontal gyrus	All	Middle frontal gyrus	2 weeks to 10.5 months; 18 months
32	F7	Frontal	All	Inferior frontal gyrus	All	Inferior frontal gyrus	All
				Lateral orbital gyrus	6, 7.5 months; 2 years	Lateral orbitofrontal gyrus	4.5, 6, 9 months to 12 years
33	FT7	Frontal	2 weeks to 3 months	Medial and inferior temporal gyri	4 years	Middle temporal gyrus	All
		Temporal	All	Superior temporal gyrus, anterior part	3 months to 20 to 24 years	Superior temporal gyrus	All
34	T7	Temporal	All	Medial and inferior temporal gyri	All	Inferior temporal gyrus	4.5 months
				Superior temporal gyrus, central part	2 months	Middle temporal gyrus	All
						Superior temporal gyrus	2 weeks to 3 months; 10.5 months
35	TP7	Temporal	All	Medial and inferior temporal gyri	2 weeks, 3 months	Inferior temporal gyrus	2 weeks to 12 years
				Posterior temporal lobe	All	Middle temporal gyrus	All
36	P7	Cerebellum	2 weeks, 1 month	Cerebellum	2 weeks, 1 month	Cerebellum	2 weeks, 1 month
		Parietal	6 months, 2, 12, 20 to 24 years	Lateral remainder of occipital lobe	6 to 10.5 months; 15 months to 4 years	Inferior temporal gyrus	4.5 to 7.5 months; 12, 18 months to 4 years
		Temporal	All	Posterior temporal lobe	All	Middle occipital gyrus	10.5 to 15 months; 2, 20 to 24 years
						Middle temporal gyrus	6, 12 months; 12, 20 to 24 years
37	PO7	Cerebellum	2 weeks to 2 months; 4.5 months	Cerebellum	2 weeks, 1 month	Cerebellum	2 weeks, 1 month
		Fusiform	10.5, 15 months	Lateral remainder of occipital lobe	All	Inferior occipital gyrus	2 weeks to 4 years
		Occipital	6, 12 months; 2, 4 years			Middle occipital gyrus	2 months to 20 to 24 years
		Parietal	2 month to 20 to 24 years				
38	O1	Cerebellum	2 weeks; 1 month	Cerebellum	2 weeks	Cerebellum	2 weeks
		Fusiform	4.5, 9, 15, 18 months	Lateral remainder of occipital lobe	All	Inferior occipital gyrus	2 weeks; 1, 4.5 months
		Occipital	1 month to 20 to 24 years			Middle occipital gyrus	All
		Parietal	3, 4.5, 7.5, 18 months, 12 years				
39	FP2	Frontal	All	Anterior orbital gyrus	7.5 months; 2 years	Middle frontal gyrus	All
				Middle frontal gyrus	All	Superior frontal gyrus	1, 2 months
				Superior frontal gyrus	All		
40	AF8	Frontal	All	Anterior orbital gyrus	6 to 12 months; 18 months, 2 years	Inferior frontal gyrus	All
				Middle frontal gyrus	All	Middle frontal gyrus	2 weeks to 12 years
41	F8	Frontal	All	Inferior frontal gyrus	All	Inferior frontal gyrus	All
				Lateral orbital gyrus	4.5 months to 4 years	Lateral orbitofrontal gyrus	3 months to 12 years
42	FT8	Frontal	2 weeks to 2 months; 6 months	Medial and inferior temporal gyri	20 to 24 years	Middle temporal gyrus	3, 4.5, 9, 10.5, 15 months; 12, 20 to 24 years
		Temporal	All	Superior temporal gyrus, anterior part	3 months to 20 to 24 years	Superior temporal gyrus	All
43	T8	Temporal	All	Medial and inferior temporal gyri	All	Inferior temporal gyrus	10.5 months
						Middle temporal gyrus	All
						Superior temporal gyrus	2 weeks to 2 months; 6, 7.5 months; 4 years
44	TP8	Temporal	All	Medial and inferior temporal gyri	2 to 7.5 months	Inferior temporal gyrus	All
				Posterior temporal lobe	All	Middle temporal gyrus	All
45	P8	Cerebellum	2 weeks to 2 months	Cerebellum	2 weeks, 1 month	Cerebellum	2 weeks, 1 month
		Fusiform	15 months	Lateral remainder of occipital lobe	10.5, 15, 18 months; 20 to 24 years	Inferior temporal gyrus	6 to 12 months; 18 months to 4 years
		Parietal	6 months; 4 to 20 to 24 years	Posterior temporal lobe	All	Middle occipital gyrus	20 to 24 years
		Temporal	All			Middle temporal gyrus	2 months; 6 to 12 months; 18 months to 20 to 24 years
46	PO8	Cerebellum	2 weeks to 2 months	Cerebellum	2 weeks, 1 month	Cerebellum	2 weeks, 1 month
		Fusiform	9, 10.5, 15, 18 months	Lateral remainder of occipital lobe	All	Inferior occipital gyrus	2 weeks to 2 years
		Occipital	12 months; 2, 4, 12 to 24 years			Middle occipital gyrus	1 month to 20 to 24 years
		Parietal	3 to 12, 18 months to 20 to 24 years				
47	O2	Cerebellum	2 weeks to 2 months	Lateral remainder of occipital lobe	All	Inferior occipital gyrus	2 weeks; 1, 4.5 months
		Fusiform	9, 10.5, 15, 18 months			Middle occipital gyrus	All
		Occipital	All				
		Parietal	3, 4.5, 7.5 months				
48	AF3	Frontal	All	Middle frontal gyrus	All	Middle frontal gyrus	All
				Superior frontal gyrus	All		
49	AF4	Frontal	All	Middle frontal gyrus	All	Middle frontal gyrus	All
				Superior frontal gyrus	All	Superior frontal gyrus	2 weeks to 2 months; 6 months
50	F5	Frontal	All	Inferior frontal gyrus	All	Inferior frontal gyrus	All
				Middle frontal gyrus	All	Middle frontal gyrus	All
51	F3	Frontal	All	Middle frontal gyrus	All	Inferior frontal gyrus	2 weeks; 4.5 to 12 months; 4 years
						Middle frontal gyrus	All
52	F1	Frontal	All	Middle frontal gyrus	2 weeks to 12 years	Middle frontal gyrus	All
				Superior frontal gyrus	All	Superior frontal gyrus	2 weeks to 4.5 months; 7.5 months to 2 years; 12, 20 to 24 years
53	F2	Frontal	All	Middle frontal gyrus	2 weeks to 4 years	Middle frontal gyrus	All
				Superior frontal gyrus	All	Superior frontal gyrus	All
54	F4	Frontal	All	Middle frontal gyrus	All	Middle frontal gyrus	All
55	F6	Frontal	All	Inferior frontal gyrus	All	Inferior frontal gyrus	All
				Middle frontal gyrus	All	Middle frontal gyrus	All
56	FC5	Frontal	All	Inferior frontal gyrus	All	Inferior frontal gyrus	All
				Precentral gyrus	All	Precentral gyrus	All
57	FC3	Frontal	All	Middle frontal gyrus	All	Middle frontal gyrus	All
				Precentral gyrus	2 weeks to 4 years	Precentral gyrus	2 weeks to 12 years
58	FC1	Frontal	All	Middle frontal gyrus	2 weeks; 2 months to 20 to 24 years	Middle frontal gyrus	All
				Precentral gyrus	2 weeks; 1 month	Precentral gyrus	2 weeks, 1 month
				Superior frontal gyrus	All	Superior frontal gyrus	All
59	FC2	Frontal	All	Middle frontal gyrus	1 month to 20 to 24 years	Middle frontal gyrus	1 month to 20 to 24 years
				Precentral gyrus	2 weeks	Superior frontal gyrus	All
				Superior frontal gyrus	All		
60	FC4	Frontal	All	Middle frontal gyrus	All	Middle frontal gyrus	All
				Precentral gyrus	2 weeks to 2 months; 10.5, 12, 15 months	Precentral gyrus	2 weeks to 2 months; 9, 12, 15 months; 12 years
61	FC6	Frontal	All	Inferior frontal gyrus	All	Inferior frontal gyrus	All
				Precentral gyrus	2 weeks to 2 months; 9 months to 20 to 24 years	Precentral gyrus	2 weeks to 2 months; 9 months to 20 to 24 years
62	C5	Parietal	All	Postcentral gyrus	3 months to 20 to 24 years	Postcentral gyrus	4.5 months to 20 to 24 years
		Temporal	2 weeks to 4 years	Remainder of parietal lobe (including supramarginal and angular gyrus)	1, 10.5, 15 months; 2, 4, 20 to 24 years	Superior temporal gyrus	2 weeks to 4 years
				Superior temporal gyrus, central part	3 to 9 months; 12 months to 4 years	Supramarginal gyrus	15 months, 20 to 24 years
63	C3	Frontal	3, 6 to 10.6, 18 months; 12, 20 to 24 years	Postcentral gyrus	All	Postcentral gyrus	All
		Parietal	All	Precentral gyrus	3, 6, 7.5 months; 12 years	Precentral gyrus	3 to 10.5 months; 12 years
				Remainder of parietal lobe (including supramarginal and angular gyrus)	2 weeks to 4 years, 20 to 24 years	Supramarginal gyrus	2 weeks to 4.5 months; 7.5, 9, 12 months to 4 years
64	C1	Frontal	All	Postcentral gyrus	2 weeks to 4.5, 7.5 months to 4 years	Postcentral gyrus	2 weeks to 2, 7.5, 12 months to 4 years
		Parietal	2 weeks to 4 years	Precentral gyrus	All	Precentral gyrus	All
				Superior parietal gyrus	1 month	Superior parietal gyrus	1 month
65	C2	Frontal	All	Postcentral gyrus	2 weeks to 2 months; 12, 15 months	Postcentral gyrus	2 weeks; 1, 15 months
		Parietal	2 weeks to 3 months; 12, 15 months	Precentral gyrus	All	Precentral gyrus	All
						Superior frontal gyrus	4.5, 6, 9 months
						Superior parietal gyrus	2 weeks
66	C4	Frontal	2 weeks; 2 months to 20 to 24 years	Postcentral gyrus	All	Postcentral gyrus	All
		Parietal	All	Precentral gyrus	3 to 10.5 months; 18 months to 20 to 24 years	Precentral gyrus	2 months to 20 to 24 years
				Remainder of parietal lobe (including supramarginal and angular gyrus)	2 weeks to 2, 15 months	Supramarginal gyrus	2 weeks to 2 months
67	C6	Parietal	All	Postcentral gyrus	2 months to 20 to 24 years	Postcentral gyrus	3 to 20 to 24 years
		Temporal	2 weeks to 12 years	Remainder of parietal lobe (including supramarginal and angular gyrus)	12 years; 20 to 24 years	Superior temporal gyrus	2 weeks to 2 years; 20 to 24 years
				Superior temporal gyrus, central part	1 months to 2 years	Supramarginal gyrus	20 to 24 years
68	CP5	Parietal	All	Posterior temporal lobe	All	Angular gyrus	4.5, 9 months to 20 to 24 years
		Temporal	2 weeks to 12 years	Remainder of parietal lobe (including supramarginal and angular gyrus)	All	Middle temporal gyrus	2 weeks to 2 months, 4 years
						Superior temporal gyrus	6, 7.5, 12, 18 months to 4 years
						Supramarginal gyrus	6, 7.5, 10.5, 12, 18 months; 12, 20 to 24 years
69	CP3	Parietal	All	Remainder of parietal lobe (including supramarginal and angular gyrus)	All	Angular gyrus	All
						Supramarginal gyrus	2 months to 20 to 24 years
70	CP1	Parietal	All	Remainder of parietal lobe (including supramarginal and angular gyrus)	2 weeks	Postcentral gyrus	10.5 months; 20 to 24 years
				Superior parietal gyrus	All	Superior parietal gyrus	All
71	CP2	Parietal	All	Postcentral gyrus	6 months	Postcentral gyrus	6 months
				Remainder of parietal lobe (including supramarginal and angular gyrus)	1 month	Superior parietal gyrus	All
				Superior parietal gyrus	All		
72	CP4	Parietal	All	Remainder of parietal lobe (including supramarginal and angular gyrus)	All	Angular gyrus	2 weeks to 4.5 months; 7.5 months to 20 to 24 years
						Supramarginal gyrus	1 month to 20 to 24 years
73	CP6	Parietal	All	Posterior temporal lobe	2 weeks to 12 years	Middle temporal gyrus	2 weeks; 1 month
		Temporal	2 weeks to 12 years	Remainder of parietal lobe (including supramarginal and angular gyrus)	All	Superior temporal gyrus	2 weeks to 12 years
						Supramarginal gyrus	2 months to 20 to 24 years
74	P5	Parietal	All	Lateral remainder of occipital lobe	2 weeks to 4 years; 20 to 24 years	Angular gyrus	2 months to 20 to 24 years
				Posterior temporal lobe	2 weeks to 7.5 months; 12, 18 months, 4 years	Middle occipital gyrus	All
				Remainder of parietal lobe (including supramarginal and angular gyrus)	2 months to 20 to 24 years		
75	P3	Parietal	All	Lateral remainder of occipital lobe	2 weeks to 4 years	Angular gyrus	2 months to 20 to 24 years
				Remainder of parietal lobe (including supramarginal and angular gyrus)	2 months to 20 to 24 years	Middle occipital gyrus	2 weeks to 9, 12 months to 4 years
76	P1	Parietal	All	Lateral remainder of occipital lobe	2 weeks to 4.5 months; 9, 12, 15 months, 4 years	Middle occipital gyrus	2 weeks; 1 month
				Superior parietal gyrus	2 monthss to 20 to 24 years	Superior occipital gyrus	2 weeks; 1 month
						Superior parietal gyrus	2 months to 20 to 24 years
77	P2	Parietal	All	Lateral remainder of occipital lobe	2 weeks to 4.5 months; 9, 12, 15 months	Angular gyrus	12 months; 2 years
				Remainder of parietal lobe (including supramarginal and angular gyrus)	2 years	Middle occipital gyrus	2 weeks; 1 month
				Superior parietal gyrus	2 weeks; 2 months to 20 to 24 years	Superior occipital gyrus	2 weeks; 1 month
						Superior parietal gyrus	2 months to 20 to 24 years
78	P4	Parietal	All	Lateral remainder of occipital lobe	2 weeks to 4.5 months; 9 to 18 months	Angular gyrus	All
				Remainder of parietal lobe (including supramarginal and angular gyrus)	All	Middle occipital gyrus	2 weeks to 3, 15 months
79	P6	Parietal	All	Lateral remainder of occipital lobe	2 weeks to 4.5 months; 9 months to 4 years; 20 to 24 years	Angular gyrus	2 months to 20 to 24 years
				Posterior temporal lobe	2 weeks to 7.5 months; 10.5 months to 2 years	Middle occipital gyrus	2 weeks to 4.5 months; 7.5 months to 4 years
				Remainder of parietal lobe (including supramarginal and angular gyrus)	2 months to 20 to 24 years		
80	PO3	Occipital	All	Lateral remainder of occipital lobe	All	Middle occipital gyrus	All
		Parietal	2 months to 20 to 24 years			Superior occipital gyrus	2 months to 20 to 24 years
81	PO4	Cerebellum	2 weeks, 2 months	Lateral remainder of occipital lobe	All	Middle occipital gyrus	All
		Occipital	All			Superior occipital gyrus	4.5 to 7.5 months; 10.5, 15, 18 months; 4 to 20 to 24 years
		Parietal	2 months to 20 to 24 years				

The Hammer and LPBA40 atlases have smaller structural segmentations than the lobar atlas. Hence, most of the channel locations were mapped to more than one ROI. Seventy-four pairs of channel-to-Hammer-ROI mapping across 67 channel locations were consistent across all age groups. Sixty-seven pairs of channel-to-LPBA40-ROI mapping across 60 channel locations were identical for all ages. Many channels had at least one identical ROI mapped to the location for all age groups across both atlases. These included channels that were sensitive to the superior frontal gyrus (FPZ, AFZ, FZ, FCZ, F2, FC1, and FC2), middle frontal gyrus (FP1, FP2, AF3, AF4, F5, F3, F4, F6, FC3, and FC4), inferior frontal gyrus (F7, F8, F5, F6, FC5, and FC6), precentral gyrus (FC5, C1, and C2), postcentral gyrus (C3 and C4), superior parietal gyrus (CPZ, CP1, and CP2), angular gyrus (CP3 and P4), cuneus (POZ), inferior temporal gyrus (FT9, T9, and T10), middle temporal gyrus (T7 and T8), and cerebellum (TP9, P9, PO9, I1, TP10, P10, PO10, and I2) across all age groups. However, between-age differences in channel-to-ROI correspondence were also observed for Hammer and LPBA40 ROI parcellations. Fourteen channel locations did not consistently map to a Hammer ROI across age groups. There were 21 channels with no consistent channel-to-LPBA40-ROI mapping across age groups.

**Fig. 9:**
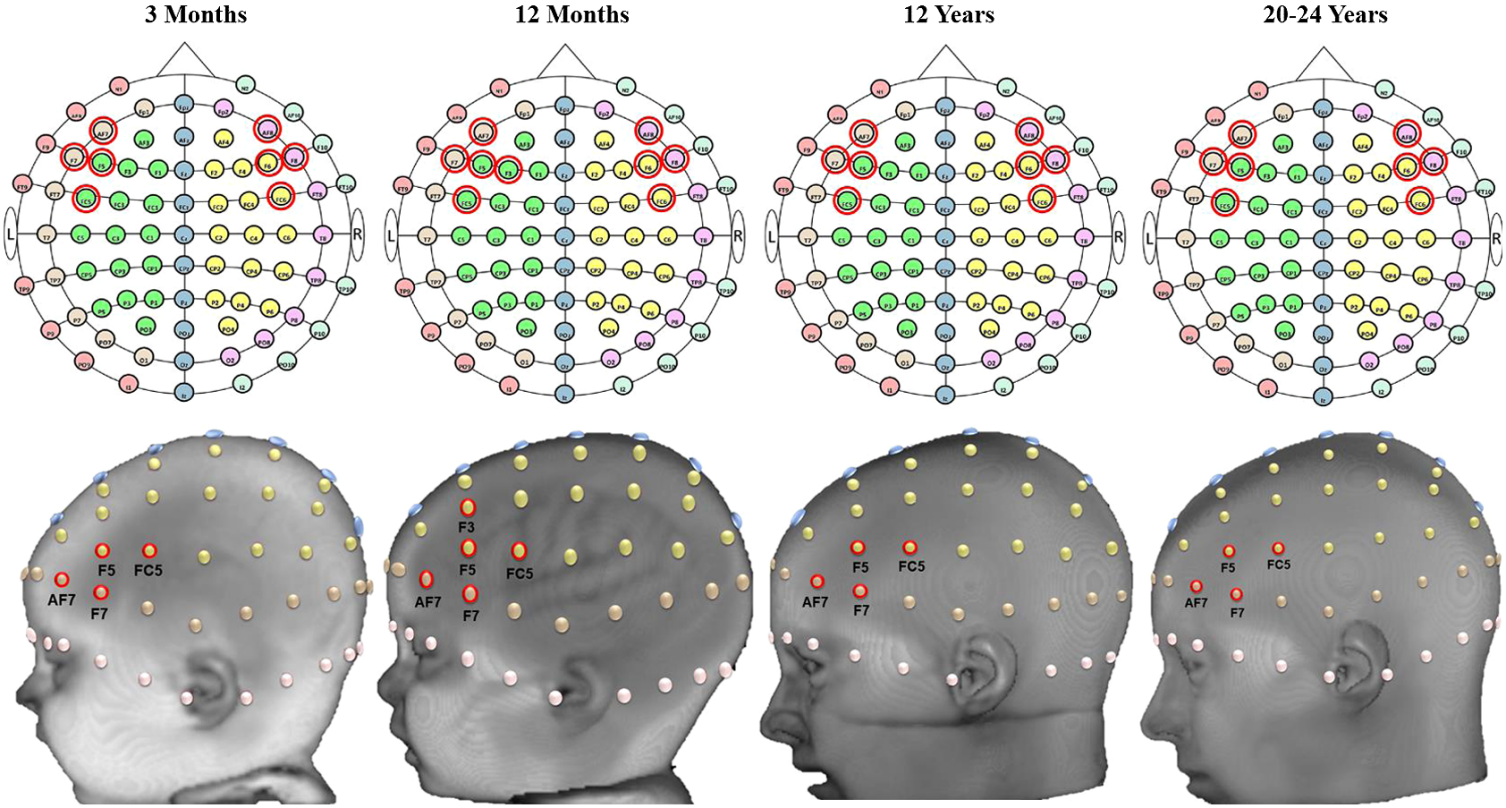
S-D channel locations sensitive to the inferior frontal gyrus. The left inferior frontal gyrus is shown on the head models. Examples of the scalp-to-ROI mapping using S-D channel DOT estimation were displayed in selected age groups (3 months, 12 months, 12 years, and 20 to 24 years). ROIs are delineated using the LONI Probabilistic Brain Atlas (LPBA40).^62^ Channel locations are displayed on age-matched average templates.

**Fig. 10:**
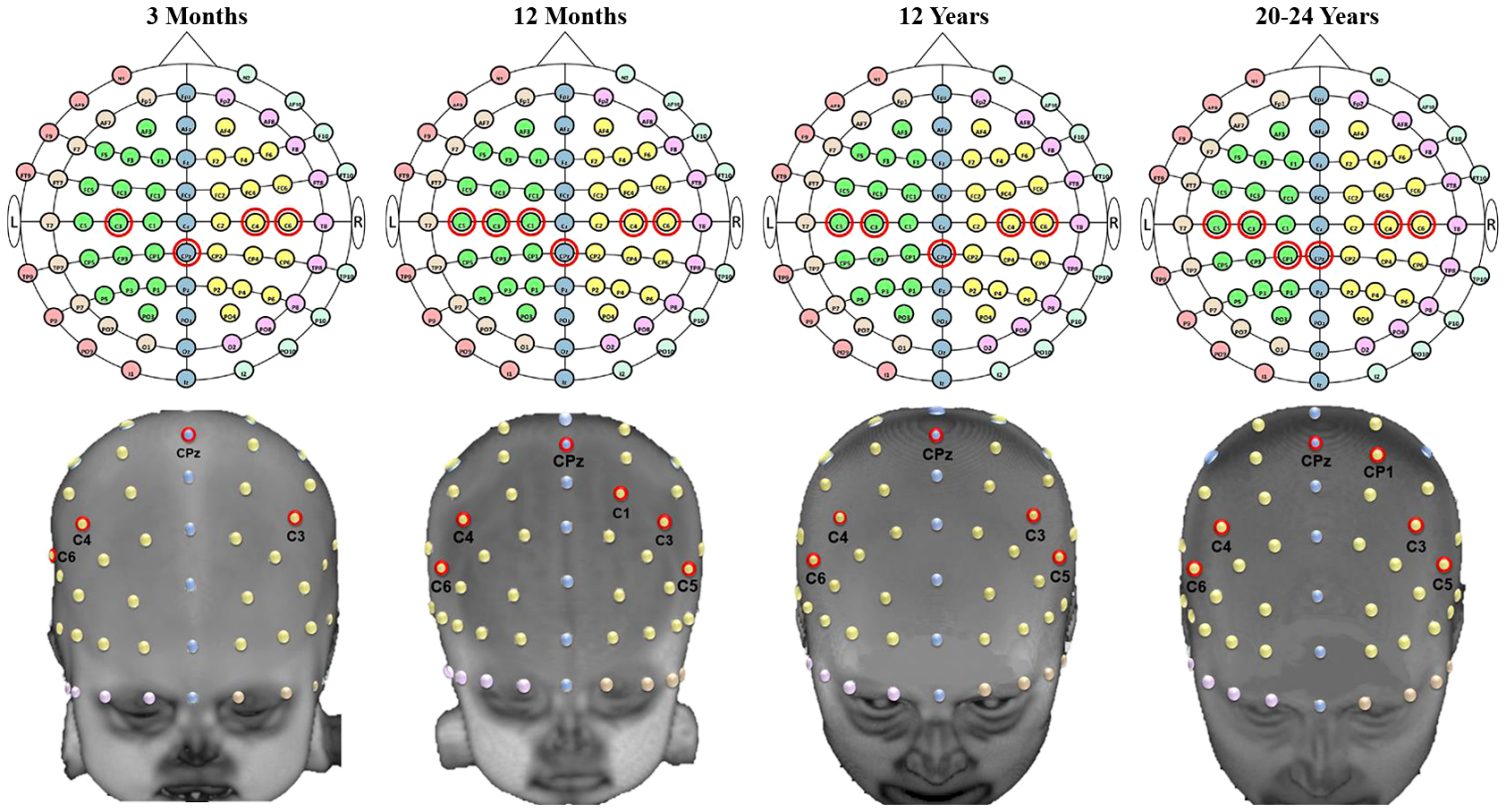
S-D channel locations sensitive to the postcentral gyrus. Examples of the scalp-to-ROI mapping using S-D channel DOT estimation were displayed in selected age groups (3 months, 12 months, 12 years, and 20 to 24 years). ROIs are delineated using the LONI Probabilistic Brain Atlas (LPBA40).^62^ Channel locations are displayed on age-matched average templates.

Figures 9 and 10 provide examples of the channel locations sensitive to ROIs delineated using LPBA40. Figure 9 shows a consistent pattern of channel locations across age groups for the inferior frontal gyrus, AF7, F5, F7, FC5, AF8, F6, F8, and FC6; channel location F3 also was sensitive to the inferior frontal gyrus at age 12 months. Figure 10 shows a less consistent pattern of channel locations sensitive to the postcentral gyrus, including CPz, CP1, C1, C3, C4, C5, and C6. However, the pattern of sensitive channel locations was different for each age.

## 4 Discussion

This study examined age differences in scalp-to-cortex distances and scalp-location-to-ROI correspondence across infancy (2 weeks to 24 months) and compared these measures to child (4, 12 years) and adult age groups (20 to 24 years). We extended existing coregistration and photon migration simulation methods to realistic head models. The use of DOT sensitivity estimated from photon migration simulations to depict scalp-to-cortex correspondence has direct applications for NIRS and fNIRS research. Our S-D channel DOT estimations revealed the scalp-to-cortex distances and underlying brain regions that are measurable with NIRS instruments across age groups. All three estimation methods showed that the scalp-to-cortex distances increased from infancy to childhood and adulthood. There were considerable variations in the distance measures among the infant age groups. The probabilistic mappings between the scalp locations and cortical lobes were relatively stable across development. However, the mappings between the scalp locations and cortical ROIs in sublobar atlas parcellations showed greater age-related variations. We found that individual participant MRIs and average MRI templates from the Neurodevelopmental MRI Database^26^^–^^29^ were similar in their scalp-to-cortex distances. This study has the key implication of highlighting the importance of using age-appropriate realistic head models to estimate DOT sensitivity that can be used to facilitate anatomical interpretations of NIRS and fNIRS data.

### 4.1 Scalp-to-Cortex Distance

Three methods were used to measure scalp-to-cortex distances. The scalp projection method has been commonly adopted in developmental and adult studies to measure the anatomical distance between the scalp electrode locations and the cortical surface.^13^^,^^17^^–^^20^ We also estimated the distance between the scalp location and the maximum fluence for the “direct DOT” fluence distribution. The distances for these two measures were very similar. This is likely due to the monotonic and somewhat nonlinear photon decay distribution across the head which is maximal near the surface of the cortex.^37^ The S-D channel DOT fluence distances were greater than distances estimated from the other methods across scalp locations for all age groups. The S-D channel DOT fluence represents the flow of photons from the source to the detector^37^ and was deeper than the cortical surface (scalp projection) or the photon flow from the optode source injection point (direct DOT). However, it is important to note that spatial projection, direct DOT, and S-D channel DOT are three distinctive methods for interrogating scalp-to-cortex correspondence. The scalp projection method measures the anatomical relation between the electrode and cortical locations. The DOT sensitivity methods describe the mapping between optode or channel locations and the underlying cortical regions that can be sampled by DOT instruments.

All three estimation methods indicated that scalp-to-cortex distances averaged across the scalp locations increased from infancy to childhood and from childhood to adulthood. This finding is consistent with Ref. 17 who measured whole-brain distances from the scalp in newborns through 12-year-olds and other studies that measured scalp-to-cortex distances by electrode positions (22- to 51-year-olds,^13^ 3.4- to 16.3-week-olds,^18^ and 5- to 11-year-olds^20^). The increase in scalp-to-cortex distances from infancy to adulthood may be attributed to several types of changes in the structure of the head. These include skull thickness,^69^^,^^70^ increases in CSF volume,^16^ global brain volume and GM/WM growth,^8^^,^^9^^,^^15^^,^^16^ and cortical folding during the first 2 years.^71^ Brain structural growth is more gradual during childhood and adolescence and reaches a plateau during adulthood.^8^^,^^9^^,^^72^^,^^73^

There were between-electrode variations in scalp-to-cortex distance, and these variations were heterogeneous among infant groups. The scalp projection and S-D channel DOT distances for all age groups were greatest at the frontal and central electrodes on the bottom row around the face area, followed by electrode locations at the midline along the intrahemispherical fissure, and the smallest on the electrode locations internal to these edges. Such interelectrode variations were also found in previous studies.^13^^,^^18^^,^^20^ Furthermore, we extended prior findings with young infants^18^ and revealed that there was a prominent decrease in distances from anterior to posterior locations in most of the infant groups (scalp projection distances in all infant ages and S-D channel DOT distances in 3-month to 15-month-olds). This anterior-to-posterior decrease in distance was less discernible in older age groups including adults measured using scalp projection.^13^ The anterior–posterior gradient coincides with the posterior-to-anterior sequence of cortical maturation.^73^^,^^74^ The occipital and parietal lobes show faster growth in volume during infancy than the frontal regions.^74^ This implies that the posterior regions have expanded closer to the scalp surface earlier than anterior regions. The region-specific heterogeneous patterns of brain development that are observable from infancy^15^^,^^16^^,^^74^^–^^76^ may contribute to the lack of systematic age-related increase in scalp-to-cortex distances among infant groups.

Hemispheric asymmetry in scalp-to-cortex distances was also observed. The spatial projection estimation showed that the scalp-to-cortex distances were larger in the right than the left hemisphere across age groups. This was consistent with the published finding and could be attributed to cortical morphometry.^17^ Greater growth in the left hemisphere may have reduced the distance between the skull and cortex surface. In contrast, the S-D channel DOT estimation revealed greater distances in the left than the right hemisphere for some infant groups, as well as children and adults. One possible interpretation is that greater S-D channel DOT fluence strength might be carried deeper into the cortex in the left than right hemisphere in these age groups. An in-depth investigation into the estimation-method-related differences in hemispheric asymmetry is outside the scope of this study. Our findings highlight that the scalp-to-cortex distances are sensitive to the complex age- and region-dependent cortical growth. Further investigations are needed to assess the hemispheric and regional differences in scalp-to-brain distances using the DOT sensitivity methods.

Gauging scalp-to-cortex distance is a foundational step for optimizing DOT sensitivity to the target cortical regions. Fu and Richards^37^ showed that the infant groups displayed different DOT sensitivity profiles compared with the adults with source–detector channels placed at the same distances. A common practice is to use longer separations for adults than infants.^77^^,^^78^ Increasing the separation distance allows the fluence distribution to extend deeper into the head tissues and thus sample cortical regions at greater depth.^35^^,^^37^ However, the increased depth sensitivity is at the expense of decreased signal strength.^79^ The current findings on age differences in the scalp-to-cortex distance can be used with age-specific DOT sensitivity profiles^37^ to find optimal source–detector separation distances that allow for comparable depth sensitivity for different age groups.

Age-appropriate MRI templates may be used to describe cranio-cerebral correspondence when subjects’ own MRIs are unavailable. This study showed that the age-appropriate templates have comparable S-D channel DOT distances as the individual head models. Furthermore, the S-D channel DOT distance estimations were robust across different Monte Carlo simulation methods (MCX, MMC, and tMCimg) for 3-month and 6-month infants’ individual MRIs and age-appropriate average templates (Figs. S5–S7 in the Supplemental Material). The developmental differences in scalp-to-cortex distances suggest that adult head models should not be used to make anatomical inferences for infant or child standalone NIRS/fNIRS data. We recommend using age-appropriate average templates or the individual MRIs when the subject’s own MRIs are not collected.^19^^,^^32^^,^^33^

### 4.2 Scalp-Location-to-ROI Mapping

This is the first study that provided scalp-location-to-ROI look-up tables (see Supplemental Table S3) computed using multiple methods for a wide range of age groups. The full look-up tables provide references for NIRS/fNIRS researchers to design optode configurations based on their study-specific age groups and ROIs. The tables can also be used to evaluate channel-to-ROI mapping for existing data. In addition, the channel-to-ROI mapping estimations can be read into programs or toolboxes to provide a user-friendly and customizable tool for optode placement optimization. The application of our estimations is further discussed below.

We used the scalp projection and the S-D channel DOT fluence to map 10–10 scalp electrode/channel locations with underlying ROIs from a macrostructural atlas (lobar) and two sublobar atlases (Hammers and LPBA40). The spatial projection method mapped underlying ROIs based only on the spatial anatomical relations. The S-D channel DOT method mapped underlying ROIs based on the DOT sensitivity profile. The S-D channel-DOT look-up table supported the prior finding that there is consistent correspondence between the majority of scalp locations and macrostructural ROIs for infants^18^^,^^25^ and adults.^13^^,^^14^ For smaller sublobar atlas ROIs, some channel locations show consistent correspondence between scalp location and underlying cortical ROI. For example, F5 and F6 were corresponded to bilateral inferior and middle frontal gyrus in all age groups. However, many channel locations show inconstant mappings across age groups. These include posterior midline positions and frontal channels on the bottom row where the scalp-to-cortex distance was larger. For example, channel TP7 was sensitive to both the inferior and middle temporal gyrus in the LPBA40 atlas for age 2 weeks through 12 years whereas it was mapped to only the middle temporal gyrus for 20- to 24-year-olds. Hemispheric symmetry/asymmetry in the mapping between channel locations and sublobar atlas ROIs was also observed. For example, the channel locations that were sensitive to the postcentral gyrus were symmetric between hemispheres at age 2 weeks, 1 month, 4.5 month, 9 month, 15 months, and 12 years but showed asymmetry for the other age groups. The observed age-related changes in hemispheric asymmetry might be related to asymmetry in the GM growth across development.^74^ Our findings further underscored the importance of using age-appropriate head models to fully account for the age-dependent head and cortical structural changes when examining craniocerebral correspondence.

The age-appropriate S-D channel DOT look-up procedures can be adopted to localize the ROI(s) that generate the fNIRS activities and design the optode arrangement prior to data collection. Our findings highlight a recognized issue in fNIRS data interpretation: the channel(s) that show significant activations from the group analysis may not correspond to the same ROI for all participants.^80^ This problem is especially concerning for infant and child studies that encompass a wide age range. Our S-D channel DOT look-up procedures provide an effective solution. The optode locations recorded from an experiment can be coregistered with individual head models from infants closely matched in age and head measurements if subjects’ own MRIs are not available. S-D channel DOT sensitivity is then estimated to infer the subject-specific ROI(s) that have generated the fNIRS signals. The methodological details were also presented elsewhere.^39^^–^^41^

Our method and look-up tables may also be used to design optode placement on NIRS holders that maximize channel sensitivity to hemodynamic changes of underlying ROIs. The tables we provide for the 10–10 or 10–5 recording system can be used as look-up tables for either manual construction of optode locations or used with automatic methods. We additionally provided an age-appropriate S-D channel DOT look-up table that quantifies the sensitivity of channel pairings from the fOLD toolbox^42^ to lobar and sublobar ROIs (Supplemental Table S4). The table provides the specificity (%) of the channel to the ROI out of the total S-D channel DOT sensitivity to all ROIs in the atlas. Researchers can thus design their holders to include channels that are sensitive to the user-specified ROIs with a specificity threshold.^42^ Figure S2(d) in the Supplemental Material shows an example of optode placement that includes channels sensitive to the left inferior frontal gyrus with specificity greater than 1% for 6-month infants. We have recently developed the devfOLD toolbox based on the existing fOLD toolbox to facilitate age-specific design of optode placement that maximizes channel-to-ROI sensitivity.^81^

### 4.3 Implications

The age-specific scalp-to-cortex distances and channel-to-ROI look-up tables based on the S-D channel DOT estimations can be used to guide NIRS and fNIRS channel placements and data analysis. Cross-sectional and longitudinal fNIRS studies need to ensure that age-group comparisons are made on data from sensors that sample the same ROI with comparable sensitivity to the cortex across ages. For example, for a study aiming to compare activations in the inferior frontal gyrus among 3-month, 12-month, and 12-year-old participants, Supplemental Table S3 and Fig. 11 indicate that sensor placement that covers channel locations AF7, F7, F5, F3, FC5, AF8, F8, F6, and FC6 could be used for all age groups. We also know that the average distances to the cortex from the frontal electrode locations differed by age (3 months: 8 mm; 12 months: 6 mm; 12 years: 10 mm). Hence, larger source–detector separation distances are expected for older ages. fNIRS studies conventionally use 20 to 30 mm separations for infants^77^^,^^82^ and 30 to 35 mm for children, e.g., Refs. 83 and 84. Experimenters should measure the source–detector separation distances after constructing a preliminary holder and adjust accordingly. Experimenters are not advised to compare activations at F3 channel locations between 3-month and 12-month-olds, as F3 may not sample activities from the left inferior frontal gyrus in 3-month-olds.

### 4.4 Limitations

This study did not compute the thickness of segmentation layers or scalp-to-cortex distances by ROI parcellations. This means that we cannot pinpoint the particular tissue layer(s) or cortical region(s) that may contribute to the age-related changes in scalp-to-cortex distance as done in Ref. 17. Furthermore, existing evidence indicated that DOT sensitivity as a function of source–detector separation distance was different in GM, WM, CSF, and extracerebral tissues (scalp and skull) in infants and adults.^34^^,^^35^^,^^85^^,^^86^ Future analyses that compare distances from the scalp by tissue types and ROIs could more precisely inform age-related differences in the optimal separation distances for sampling cortical signal changes at different brain regions.

Differences in data collection sites may contribute to the differences in data collection sites. We included MRIs from a wide range of data sources to increase the generalizability of our results on scalp-to-cortex correspondence. However, the number of participants in each age group was not evenly distributed across databases. This was partly due to the design and the specific age groups targeted in the individual database. Hence, we cannot tease apart the effect of age and database in this study. We examined the effect of age, estimation method, and electrode locations with databased as a categorical covariate variable. The analyses are presented in the Supplemental Material. The interaction effect of the three factors remained after controlling for database difference. However, the specific pattern of age-related differences across infant age groups varied with the covariate added to the model. With the expansion of the Neurodevelopmental MRI Database, our future study aims to balance the number of participants from each group across the databases.

## 5 Conclusions

This study examined the scalp-to-cortex distances and scalp-to-cortical ROI correspondence in infants, children, and adults using both the scalp projection methods and DOT sensitivity estimations. There were differences in the scalp-to-cortex distance in infants, which become magnified in children and adults. We found systematic differences in scalp-location-to-anatomical ROI correspondence for different ages, both for spatial projection and for DOT sensitivity. Our findings imply that accurate anatomical interpretations of NIRS/fNIRS data are dependent on developmentally sensitive estimations of DOT sensitivity that account for the head and cortical development. This study demonstrated that age-appropriate realistic head models should be used to provide anatomical guidance for standalone DOT data.

## Code, Data, and Materials Availability

The age-specific average templates, including T1, DOT sensitivity average template can be accessed from the “Neurodevelopmental MRI Database.” The database is available online: http://jerlab.psych.sc.edu/NeurodevelopmentalMRIDatabase/ for information and https://www.nitrc.org/projects/neurodevdata/for access. Details for the online access are provided in Ref. 27. The codes that support the findings of this study are available from the corresponding author, XF, upon request.
