# Supplementary material for: Investigating developmental changes in scalp-to-cortex correspondence using diffuse optical tomography sensitivity in infancy
Source: Neurophotonics. 2021 Jul 24;8(3):035003. doi: 10.1117/1.NPh.8.3.035003 (PMC8305752; doi:10.1117/1.NPh.8.3.035003)
Supplement: Supplementary file 1 [file NPh_008_035003_SD001.pdf]

Supplemental Information for "Investigating Developmental Changes in Scalp-Cortex Correspondence Using Diffuse Optical Tomography Sensitivity in Infancy"

Method

Age-appropriate MRI Average Template

Age-appropriate average template was constructed for each age group. The procedure is detailed in literatures on the Neurodevelopmental MRI Database (Fillmore, Phillips-Meek, & Richards, 2015; Fillmore, Richards, Phillips-Meek, Cryer, & Stevens, 2015; Richards, in prep; Richards, Sanchez, Phillips-Meek, & Xie, 2015; Sanchez, Richards, & Almli, 2012a, 2012b). Briefly, the average templates were created with an iterative procedure. The first step was to create a tentative average based on a rigid rotation (FLIRT 6 parameter linear registration and tranformation; Jenkinson & Smith, 2001) to the MNI-152 adult template (Joshi, Davis, Jomier, & Gerig, 2004; Mazziotta et al., 2001). The individual MRIs were then registered to this template and transformed to the template space with non-linear registration (ANTS; Avants et al., 2011), followed by averaging of the transformed MRIs. This became the reference template in the next iteration. The root mean square (RMS) difference between successive averages was computed. The iterative steps continued until the RMS between subsequent averages was minimized.

Mesh Generation

We generated both sparse and dense "segmented FEM mesh" (also see Method in the main text). The mesh is required only for the MMC package (Fang, 2010). The sparse mesh was used for the MMC when the program did not converge with the dense mesh. The dense "segmented FEM mesh" was used for MCX (Fang & Boas, 2009) and tMCimg (Boas, Culver, Stott, & Dunn, 2002) to find a segment element that is closest to an electrode position. Figure S1

shows the mean numbers of nodes and elements for sparse and dense meshes across age groups. The average number of nodes was 301263, 417523, 440384, for the infants, children, and adults, respectively; average number of elements was 1,752,221, 2,449,375, and 2,582,646; and average tetra volumes were 21, 22, and 27 cubic mm. The change in node and element size reflect increases in head size over these ages.

10-10 Electrodes Placement

The 81 10-10 electrode locations were constructed based on the "unambiguously illustrated 10-10 system (Jurcak, Tsuzuki, & Dan, 2007). Cranial fiducial points were manually placed on each MRI volume using MRIcron (Rorden, 2012; Rorden & Brett, 2000). The fiducial points were nasion (Nz), vertex (Vz), inion (Iz), left preauricular point (LPA), right preauricular point (RPA), left mastoid (LMa), and right mastoid (RMa) (Richards, Boswell, Stevens, & Vendemia, 2015). We divided the electrode positions to six groups for visualization purposes. The Cz was located at the intersection of the front-to-back central curve (Nz to Iz) and the left-to-right central curve (LPA to RPA). The "z" electrodes were placed at the 10% intervals on the Nz-Cz-Iz central curve (group 1). The LPA-RPA central curve was divided in 10% increments to set electrodes T7 to T8. From Nz to LPA to Iz, the N1, I1, and the "9" electrodes (e.g. AF9 and PO9) were identified in 10% intervals (group 2), and likewise for the N2, I2, and the "10" electrodes (e.g. AF10 and PO10) set on the right hemisphere (group 3). The curve Fpz to T7 to Oz was divided in 10% increments to generate Fp1, O1, and the "7" electrodes (group 4), and the same method was applied to identify Fp2, O2, and the "8" electrodes on the right hemisphere (group 5). Lastly, the "z", "7" and "8" locations were used to define "1" and "2", '3" and "4", and "5 and "6" electrode locations (group 6).

Virtual Channel Construction

We constructed 130 S-D channels from neighboring 10-10 electrode locations based on the fNIRS Optodes' Location Decider (fOLD) toolbox (Zimeo Morais, Balardin, & Sato, 2018). These channels were centered at the 81 10-10 electrode locations and located at single surrounding 10-10 electrode locations. The fOLD channels were formed for the individual MRIs and age-matched average templates of 3-month-olds ($M_{separation}$=23.08mm, SD=1.79), 6-month-olds ($M_{separation}$=24.41mm, SD=1.01), and 20-24-year-olds ($M_{separation}$=29.92mm, SD=1.59). Figure S2 displays the fOLD channels on the 6-month average template.

Monte Carlo (MC) Photon Migration Simulations

The inputs of Monte Carlo simulations include a segmented head model that defines the tissue types. Five tissue types are typically identified: the scalp, skull, cerebrospinal fluid (CSF), gray matter, and white matter (Fukui, Ajichi, & Okada, 2003; Mansouri, L'Huillier, Kashou, & Humeau, 2010; Strangman, Li, & Zhang, 2013). We launched 10^8 photons for the time window of 0 to 5 nanoseconds. The fluence resolution was 50 time-gates (Zimeo Morais et al., 2018). The wavelength was set at 690 nanometers. Photons were sent from the 358 10-5 electrode positions. The optical properties of the tissue types that are specified include the absorption coefficient, the scattering coefficient, the anisotropy coefficient, and the index of refraction. It should be noted that there has been no consensus on the optical properties in the literature (Zimeo Morais et al., 2018). The values differ considerably across studies (Boas et al., 2002; Brigadoi & Cooper, 2015; Custo et al., 2010; Strangman, Franceschini, & Boas, 2003; Strangman et al., 2013; Whiteman, Santosa, Chen, Perlman, & Huppert, 2017; Zimeo Morais et al., 2018). Different values may be needed for adult and infant-child participants (cf Brigadoi & Cooper, 2015; Fukui et al., 2003; Whiteman et al., 2017). Our input values for the optical properties of the head media were shown in Table S1. They were based on values used in

Perdue, Fang, & Diamond (2012), which primarily came from Strangman, Franceschini, & Boas (2003) and Yaroslavsky et al. (2002). These values were also used in Fang (2010).

We used the MC eXtreme (MCX; Fang & Boas, 2009), tMCimg (Boas DA, 2002), and a mesh-based MC method (MMC; Fang, 2010) for modeling photon propagation. The principal distinctions between tMCimg, MCX, and MMC are computational efficiency and the modeling of boundary structure. MCX improves from tMCimg by employing parallel computing in graphics processing units (GPUs) to simultaneously modeling the propagation of a large number of photons. tMCing and MCX are both voxel-based simulations. This means that they discretize boundaries into voxelated, grid-like geometry. This could introduce estimation error of reflection and transmission when a photon reaches certain boundaries. MMC mitigates the problem by using mesh-based head models with tetrahedral elements and ray-tracing calculations. It better approximates complex boundary structures and thus could provide more accurate fluence estimation (Fang, 2010; Tran & Jacques, 2020; Yan, Tran, & Fang, 2019). The input parameters for tMCimg and MMC were the same as those for MCX (see the "Photon Migration Simulations" in Method). MMC was only performed 3-month and 6-month individual MRIs and age-matched average templates.

DOT Sensitivity Analyses from tMCimg and MMC Outputs

We computed the "Direct DOT" and "S-D Channel DOT" as described in the "DOT Sensitivity Analyses" section in the Main Text. The Direct DOT distance was defined as the distance from the 10-10 scalp electrode location to the brain voxel with the maximum fluence value. The S-D Channel distance was measured as the distance from the 10-10 channel location (see "Virtual Channel Construction" in the Main Text) to the voxel with the maximum S-D Channel DOT value.

DOT Sensitivity Analyses for fOLD Channels

S-D Channel DOT was calculated for each fOLD channel by taking the product of source fluence distribution and the detector fluence distribution. We did not normalize S-D Channel DOT outputs by the sum of fluence for all voxels as described in Zimeo Morais et al. (2018). We also computed the S-D Channel distance for fOLD channels.

Results

Scalp-to-Cortex Distance

Differences in scalp-to-brain distances across electrode locations, estimation method, and age groups. Figure S3 provides visualizations of scalp-to-cortex distances by individual electrode locations estimated using Scalp projection, Direct DOT, and S-D Channel DOT for infants and toddlers, children and adults. Figure S3A that the mean distances were largest for the adults, followed by children and infants across electrode locations and estimation methods. The larger distances were at the bottom row electrode positions, followed by the midline electrodes for all estimation methods and age groups. At most electrode locations, the S-D Channel DOT distances were larger than the Scalp Projection and Direct DOT distances for all age groups. Figure S3B additionally highlights the age-group differences for the S-D Channel DOT distance measures across electrode positions. The main effects of age group, estimation method, and estimation method are detailed in the Main Text.

Sex difference in scalp-to-cortex distance estimated using S-D Channel DOT. Figure S4 presents the effects of sex, age group, and electrode group on S-D Channel DOT distances. A three-way ANOVA with sex, age group, and electrode group revealed the expected effect of age group, F (15, 4080) = 722.34, p<.001, electrode group, F (3, 4080) = 3569.87, p<.001, and age-by-electrode-group interaction effect, F (45, 4080) = 7.72, p<.001, as described in the Main Text.

Additionally, there were significant effects of sex, F (1, 4080) = 39.07, p<.001, and sex-by-age, F (15, 4080) = 2.86, p<.001. The sex-by-electrode-group and sex-by-age-by-electrode-group interaction effects were not significant. The mean S-D Channel DOT distance was greater in males than females averaged across age groups and electrode groups, p<.001. The sex difference did not emerge or change systematically across development. Males had greater distances than females at age 2 Months, 4.5 Months, 6 Months, 7.5 Months, 12 Months, 15 Months, and 20-24 Years, p<.05, but not at other ages.

Comparison of scalp-to-cortex distances across head model types and estimation methods. We compared S-D Channel DOT distance computed from the average MRI templates and participant-based individual MRIs. Figure S5 shows that the averaged distances across individual 10-10 channel positions by age group and electrode group computed from age-matched average templates (Figure S5A) and individual head models (Figure S5B). The S-D Channel DOT distances computed using average templates were similar with those calculated from the individual MRIs (c.f. Main Text Figure 4). Specifically, 1) the S-D Channel DOT distances calculated from average templates increased from infancy to childhood and to adulthood; 2) there were no systematic age-group differences among infants; and 3) the age-group differences changed across electrode locations.

Figure S6 shows a comparison between MCX versus tMCimg estimations of the Direct DOT and Channel DOT. The two methods for simulating photon migrations through the head media produced comparable results across age groups.

Figure S7 compares of S-D Channel DOT distances estimated using MCX, MMC, and tMCimg (10-10 channels) with distances estimated using MCX fOLD channels in 3-month and 6-month individual head models and age-matched average templates. MCX, MMC, and tMCimg

estimation methods produced comparable distance estimates for 3-month and 6-month individual MRIs and average templates. However, the pattern of age-group difference was dependent on the head model type. The mean S-D Channel DOT distance across fOLD channels was comparable with MCX, MMC, and tMCimg estimations for 3-month individual head models. However, the mean distance across the fOLD channels was larger than the mean distance across the 10-10 channels in the 6-month individual MRIs. The mean distance across fOLD channels in the average templates were higher than the distance across the 10-10 channels in both the average templates and individual head models.

The effect of database on scalp-to-cortex distance. We examined scalp-to-cortex distances as a function of age group (2 weeks to 20-24 years), estimation method (Scalp Projection, Direct DOT, S-D Channel DOT), and electrode group with database entered as a categorical covariate variable. The three-way ANCOVA showed a significant effect of database, F (5, 12139) = 163.59, $p < .001$, age group, F (15, 12139) = 872.42, $p < .001$, estimation method, F (2, 12139) = 1227.94, $p < .001$, and electrode group, F (3, 12139) = 15814.10, $p < .001$. The two-way interaction effects were significant: age group × estimation method, F (30, 12139) = 4.94, $p < .001$; age group × electrode group, F (45, 12139) = 19.56, $p < .001$; and estimation method × electrode group, F (6, 12139) = 114.44, $p < .001$. The age group × estimation method × electrode group interaction effect remained significant with the covariate added to the model, F (90, 12139) = 1.69, $p < .001$.

We further examined the age group and estimation method interaction effect on scalp-to-cortex distances with databased entered as a covariate. The age-related differences remained for Scalp Projection, F (15, 12139) = 265.90, $p < .001$, Direct DOT, F (15, 12139) = 568.85, $p < .001$, and S-D Channel DOT distance, F (15, 12139) = 464.58, $p < .001$, after controlling for the

effect of database. The pattern of between-age-group differences changed with the effect of databased controlled for (c.f. Main Text Figure 4). For example, results for the Scalp Projection distances showed that the difference between age 4.5 months (M = 7.78) and age 6 months (M = 7.58) was not significant, p = .15, the distance increased from age 10.5 months (M = 6.86) to 12 months (M = 7.60), p < .001, and the distance increased from age 18 months (M = 7.18) to 2 years (M = 7.65), p < .001. For the Direct DOT estimation, the distance decreased from 4.5 months (M = 6.94) to 6 months (M = 6.60), p <. 05, increased from 10.5 months (M = 6.20) to 12 months (M = 6.83), p < .001, increased from 18 months (M = 6.50) vs. 2 years (M = 6.80), p < .05. For the S-D Channel DOT estimation, the distance decreased from 4.5 months (M = 8.89) to 6 months (M = 8.30), p < .001. The distance did not significantly change from 10.5 months (M = 8.12) to 12 months (M = 8.28), p = .29, or from 18 months (M = 8.29) vs. 2 years (M = 8.43), p = .29.

Scalp-location-to-ROI Mapping

Table S3 (linked from the Main Text) provides a look-up procedure that maps the scalp electrode and channel locations to the ROIs from the lobar (Fillmore, Richards, et al., 2015), Hammer (Heckemann, Hajnal, Aljabar, Rueckert, & Hammers, 2006), and LPBA40 atlas (Shattuck et al., 2008). The spatial scalp projection was used to locate the atlas ROI(s) that intersected with the spherical mask created around the 10-10 electrode location. We also identified ROI(s) in the spherical mask that intersected with the S-D channel DOT fluence distribution (Main Text Figure 3). The percentage of voxels in the ROI is displayed in parentheses. ROIs with less than 10% voxels were not shown.

Table S4 (linked from the Main Text) provides a comparable look-up procedure as Zimeo Morais (2018). The table displays the anatomical specificity of a given fOLD channel to the

ROI(s). We divided the S-D channel DOT for an ROI by the total channel DOT for all ROIs to compute the specificity (%) to the ROI of a given channel (Zimeo Morais et al., 2018). We additional examined fOLD channel specificity to ROIs from the Brainnetome Atlas (Fan et al., 2016). The atlas provides a microanatomical parcellation of 210 cortical and 36 subcortical subregions. The table displays ROIs with channel specificity greater than or equal to 10%. Channel specificities for the Brainnetome ROIs were smaller since the atlas has more fine-grained parcellations. We listed the Brainnetome ROIs with the largest specificity for a given channel if none of the ROI exceeded the 10% specificity threshold.

Table S1. Optical properties used in Monte Carlo photon migration simulations. The wavelength was set at 690 nanometers. μ_a is the optical absorption coefficient; μ_s is the scattering coefficient, g is the anisotropy coefficient, and N is the index of refraction.

**Tissue Type**	**μ_a (mm^{-1})**	**μ_s (mm^{-1})**	***g***	***N***
White matter	0.07	40.1	0.85	1.37
Gray matter	0.02	8.4	0.90	1.37
CSF	0.0004	1	0.99	1.37
Dura	0.0101	80	0.99	1.37
Skull	0.0101	100	0.99	1.37
Skin	0.0101	80	0.99	1.37
Muscle	0.0101	80	0.99	1.37
Eyes	0.0004	1	0.99	1.37
Nasal Cavity	0.0101	80	0.99	1.37
Non-myelinated Axons	0.07	40.1	0.85	1.37

Table S2. Parameter estimates of analysis of variance (ANOVA) models performed in the Main Text.

Source of Variation	*DF*	Mean Square	*F* Value	*p* Value
**Model 1 DV: Distances (mm) from All Estimation Methods**				
Age Group	15	3646.40	1652.55	<.0001
Electrode Group (midline, lowest circumference, second-lowest circumference, remaining)	3	32704.97	14821.90	<.0001
Estimation Method	2	2539.24	1150.78	<.0001
Age × Electrode	45	40.46	18.34	<.0001
Age × Method	30	10.25	4.65	<.0001
Electrode × Method	6	236.68	107.26	<.0001
Age × Electrode × Method	90	3.50	1.59	.0004
**Model 2a DV: Scalp Projection Distances (mm)**				
Age Group	15	809.19	374.64	<.0001
Electrode Group (midline, lowest circumference, frontal, remaining)	3	13809.41	6393.45	<.0001
Age × Electrode	45	16.28	7.54	<.0001
**Model 2b DV: S-D Channel DOT Distances (mm)**				
Age Group	15	1591.74	690.39	<.0001
Electrode Group (midline, lowest circumference, frontal, remaining)	3	8110.41	3517.76	<.0001
Age × Electrode	45	29.25	12.69	<.0001
**Model 3a DV: Scalp Projection Distances (mm)**				
Age Group	15	1128.63	432.03	<.0001
Electrode Group (lowest circumference, second-lowest circumference, remaining)	2	40913.99	15661.50	<.0001
Hemisphere	1	50.54	19.35	<.0001
Age × Electrode	30	29.75	11.39	<.0001
Age × Hemisphere	15	2.53	0.97	.49
Electrode × Hemisphere	2	1.50	0.58	.56
Age × Electrode × Hemisphere	30	1.81	0.69	.90
**Model 3b DV: S-D Channel DOT Distances (mm)**				
Age Group	15	2257.44	870.31	<.0001
Electrode Group (lowest circumference, second-lowest circumference, remaining)	2	23990.22	9248.99	<.0001
Hemisphere	1	115.39	44.49	<.0001
Age × Electrode	30	38.56	14.87	<.0001
Age × Hemisphere	15	16.07	6.20	<.0001
Electrode × Hemisphere	2	8.16	3.14	.04
Age × Electrode × Hemisphere	30	1.70737	0.66	.92

Note: DV = dependent variable.

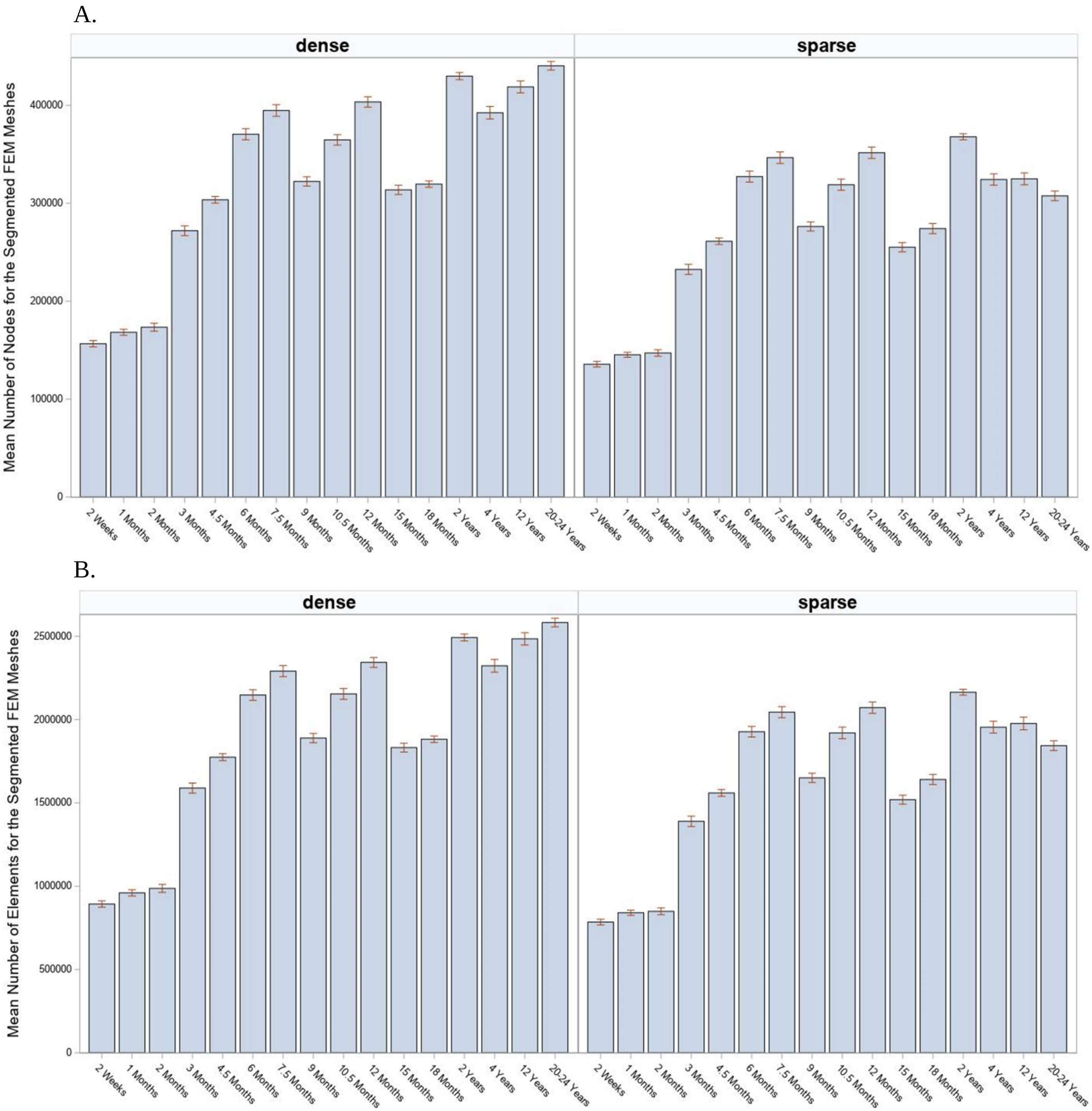

Fig S1. Mean number of nodes and elements for the dense and sparse finite element model (FEM) mesh by age groups.

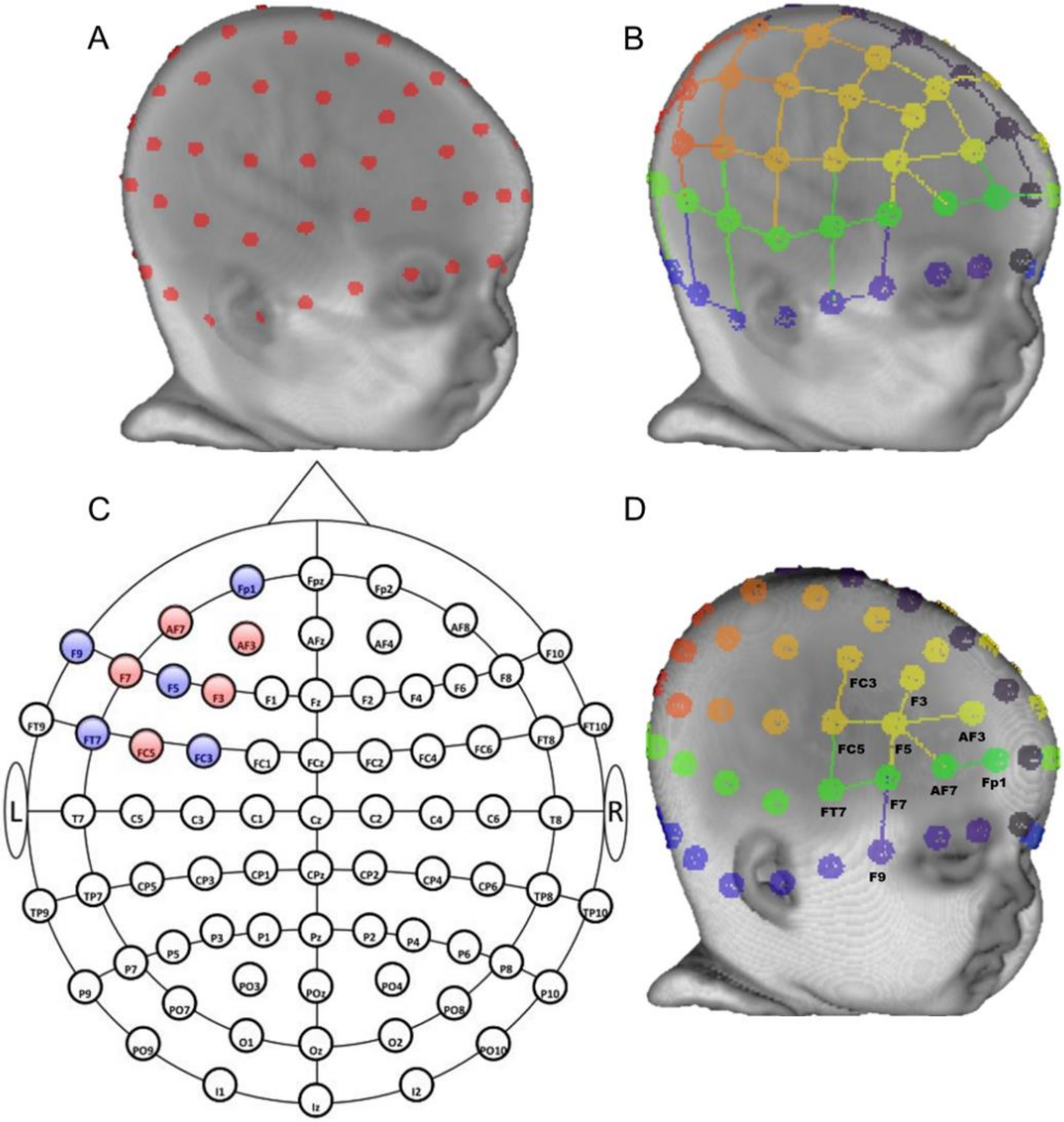

Fig S2. Virtual channel placement for fOLD channels. Channels constructed based on the fNIRS Optodes' Location Decider (fOLD) were displayed on an average template for 6-month infants. A. Ten-ten virtual electrode placement. B. fOLD channel topographical layout. C. Two-dimensional layout of the 10-10 system from the fOLD graphical user interface. It shows the source (red) and detector (blue) combination for the left inferior frontal gyrus from the LONI Probabilistic Brain Atlas (LPBA40; Shattuck et al., 2008). The specificity threshold was set at 1%. D. The topographical layout for channels that are sensitive to the left inferior frontal gyrus with specificity greater than 1%.

A.

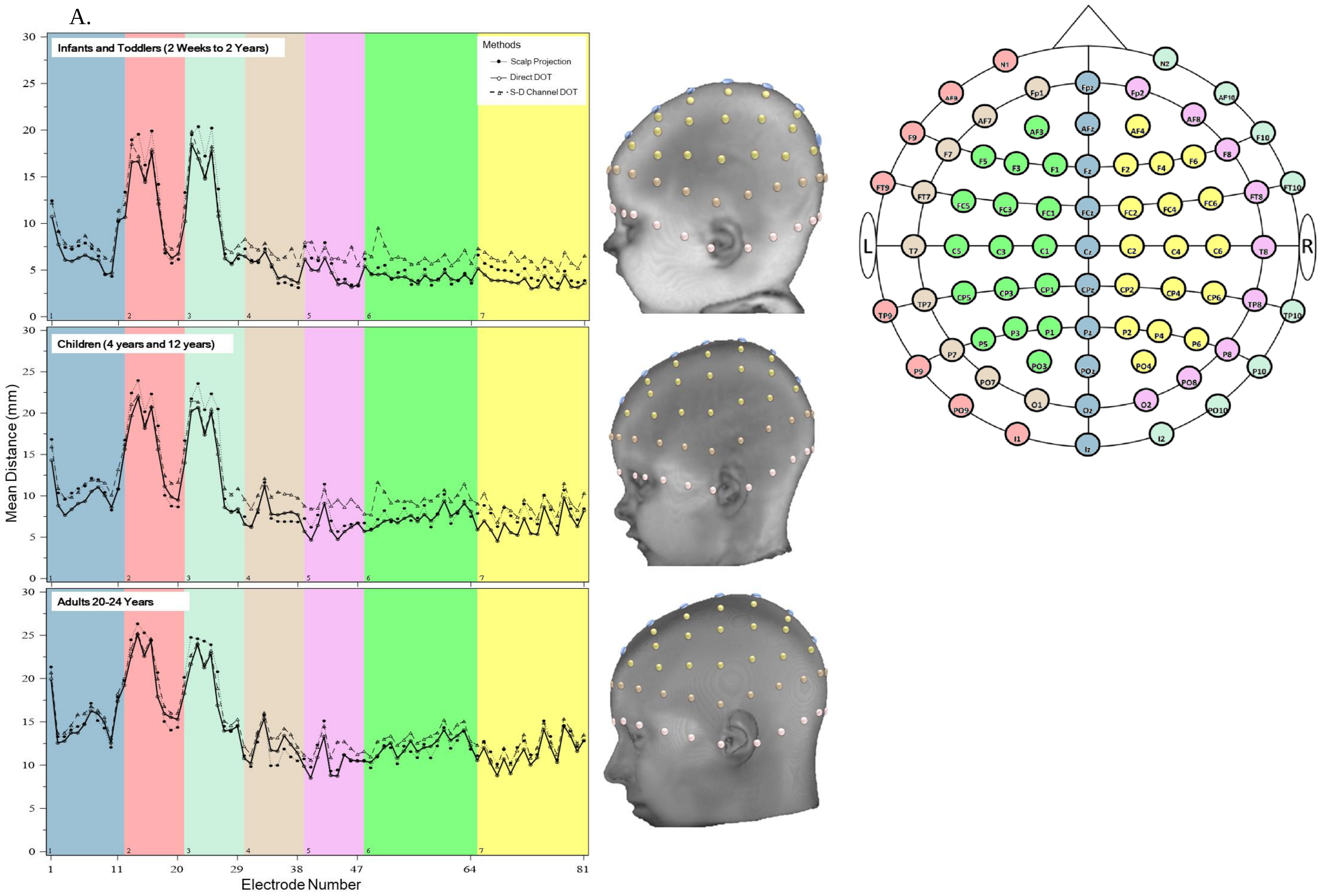
Infants and Toddlers (2 Weeks to 2 Years)
Children (4 years and 12 years)
Adults 20-24 Years
Methods
Scalp Projection
Direct DOT
S-D Channel DOT
Mean Distance (mm)
Electrode Number
L
R

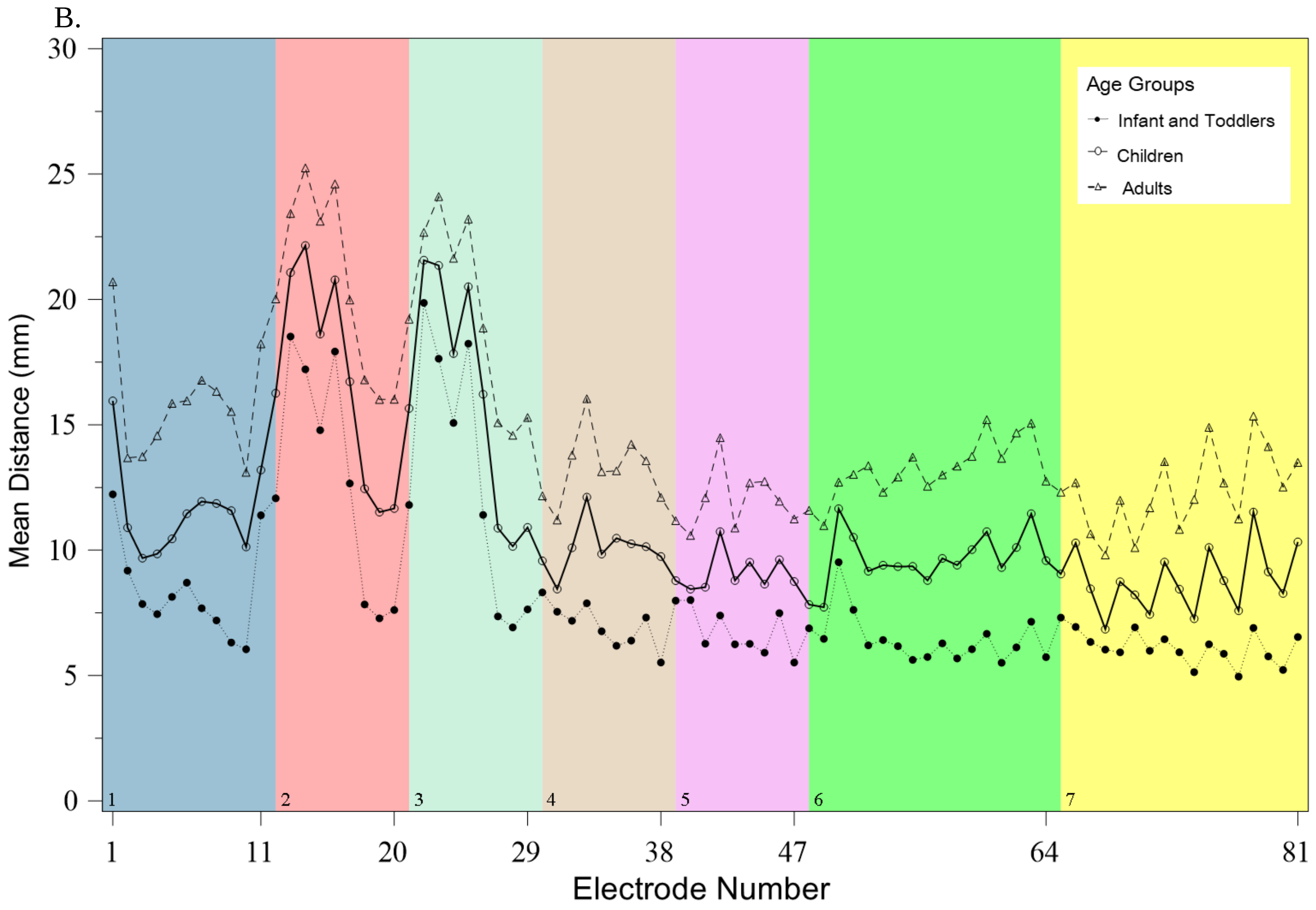

Fig S3. Mean scalp-to-cortex distances across individual 10-10 electrode/channel locations by estimation methods and age groups. A. Distances estimated using Scalp Projection, Direct DOT and S-D Channel DOT with individual MRIs. For visualization purpose, electrode positions are color-coded by circumference location and hemisphere (c.f. Main Text Figure 2). In addition, a two-dimensional layout of the 10-10 system (top right), a three-dimensional rendering of the electrode locations on a 3-month-old infant average template (top left), a 4-year-old child average template (middle) and a 20-24-year-old adult average template (bottom) were displayed to aid visualization. B. Mean S-D Channel DOT distances across individual 10-10 channel locations for infants and toddlers (2 weeks to 2 years), Children (4 years and 12 years) and adults (20-24 years).

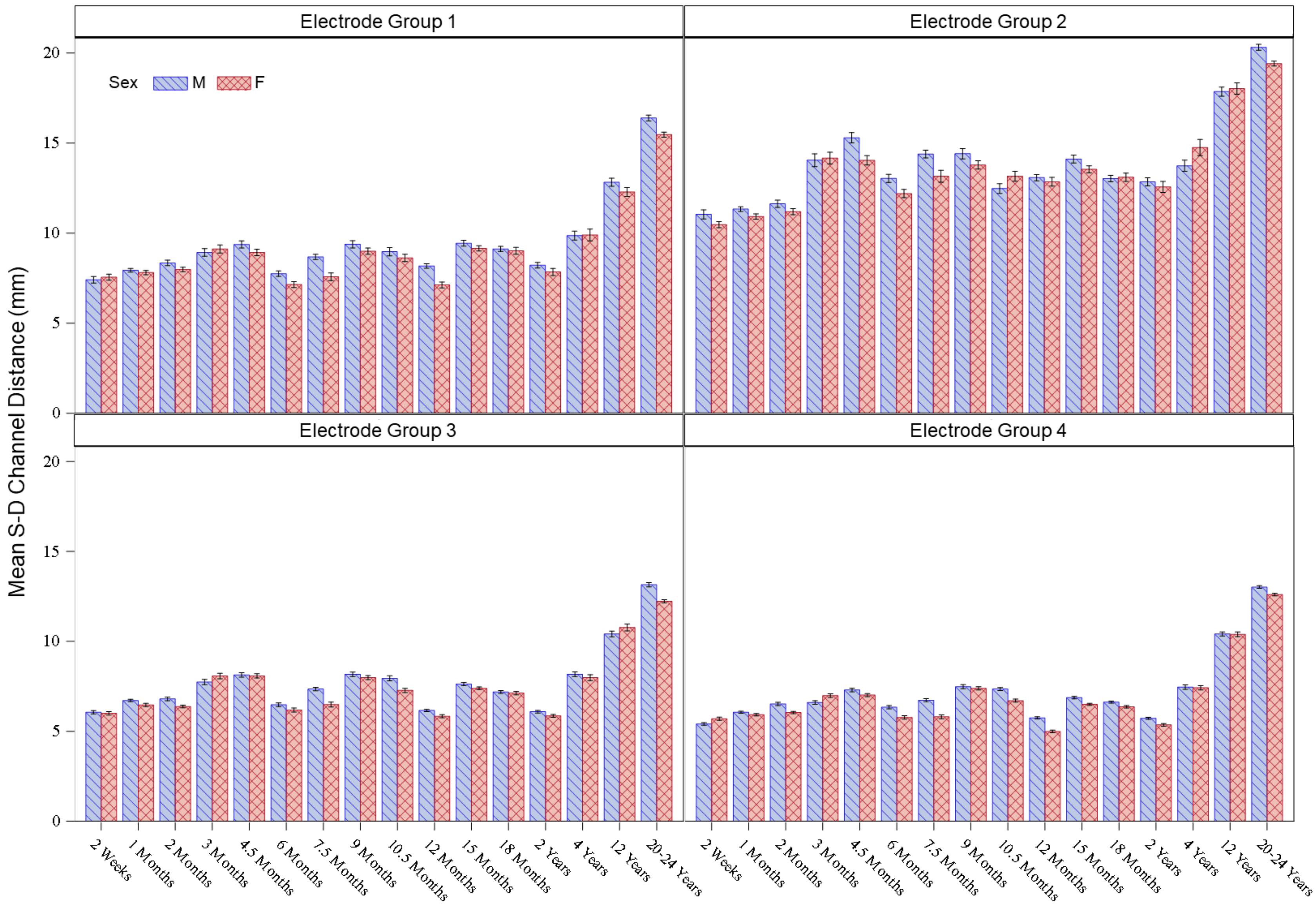

Fig S4. Mean S-D Channel DOT distances averaged across individual 10-10 channel locations by sex, age, and electrode group.

A.

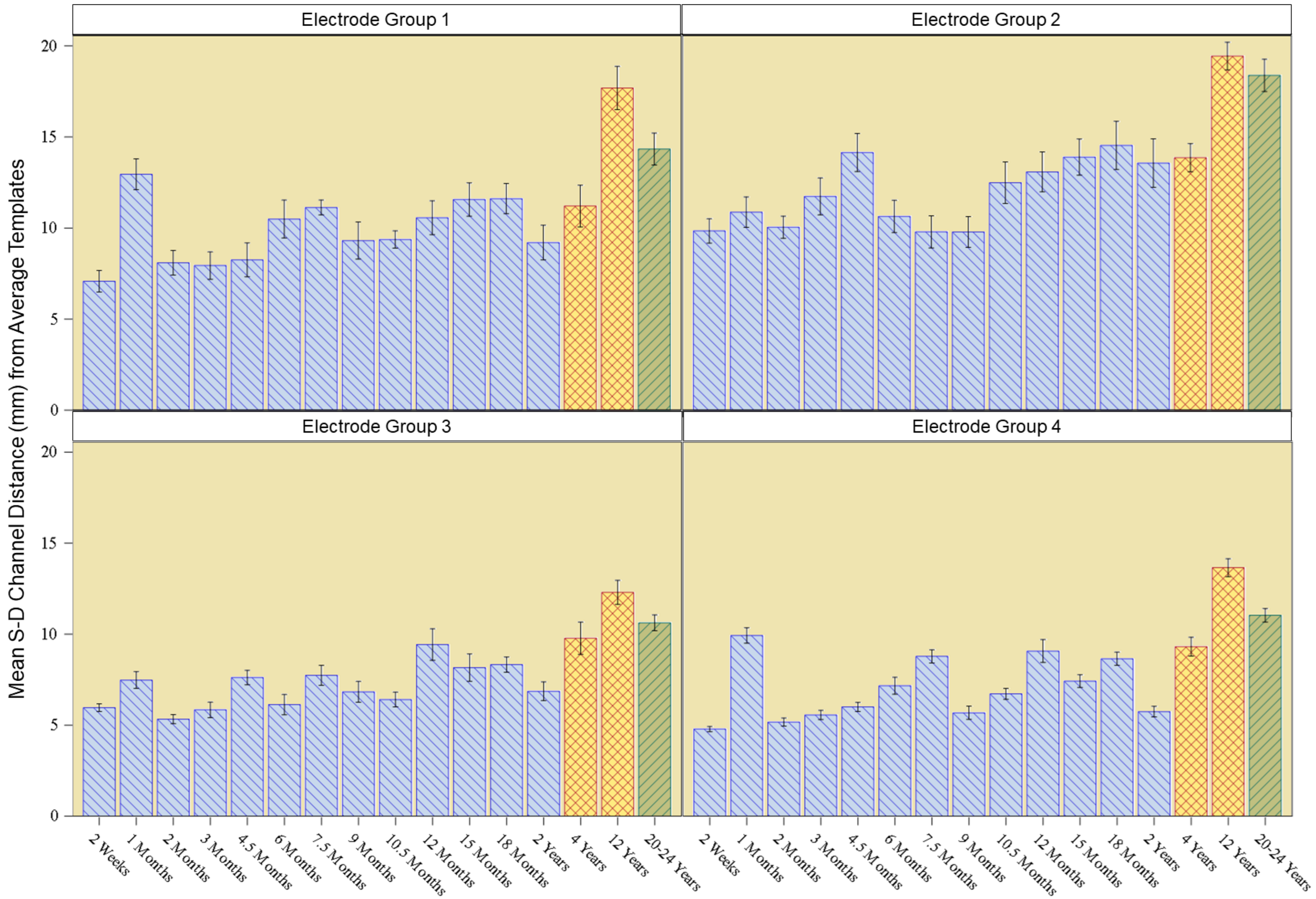

Electrode Group 1
Electrode Group 2
Electrode Group 3
Electrode Group 4
Mean S-D Channel Distance (mm) from Average Templates
0
5
10
15
20
2 Weeks
1 Months
2 Months
3 Months
4.5 Months
6 Months
7.5 Months
9 Months
10.5 Months
12 Months
15 Months
18 Months
2 Years
4 Years
12 Years
20-24 Years

B.

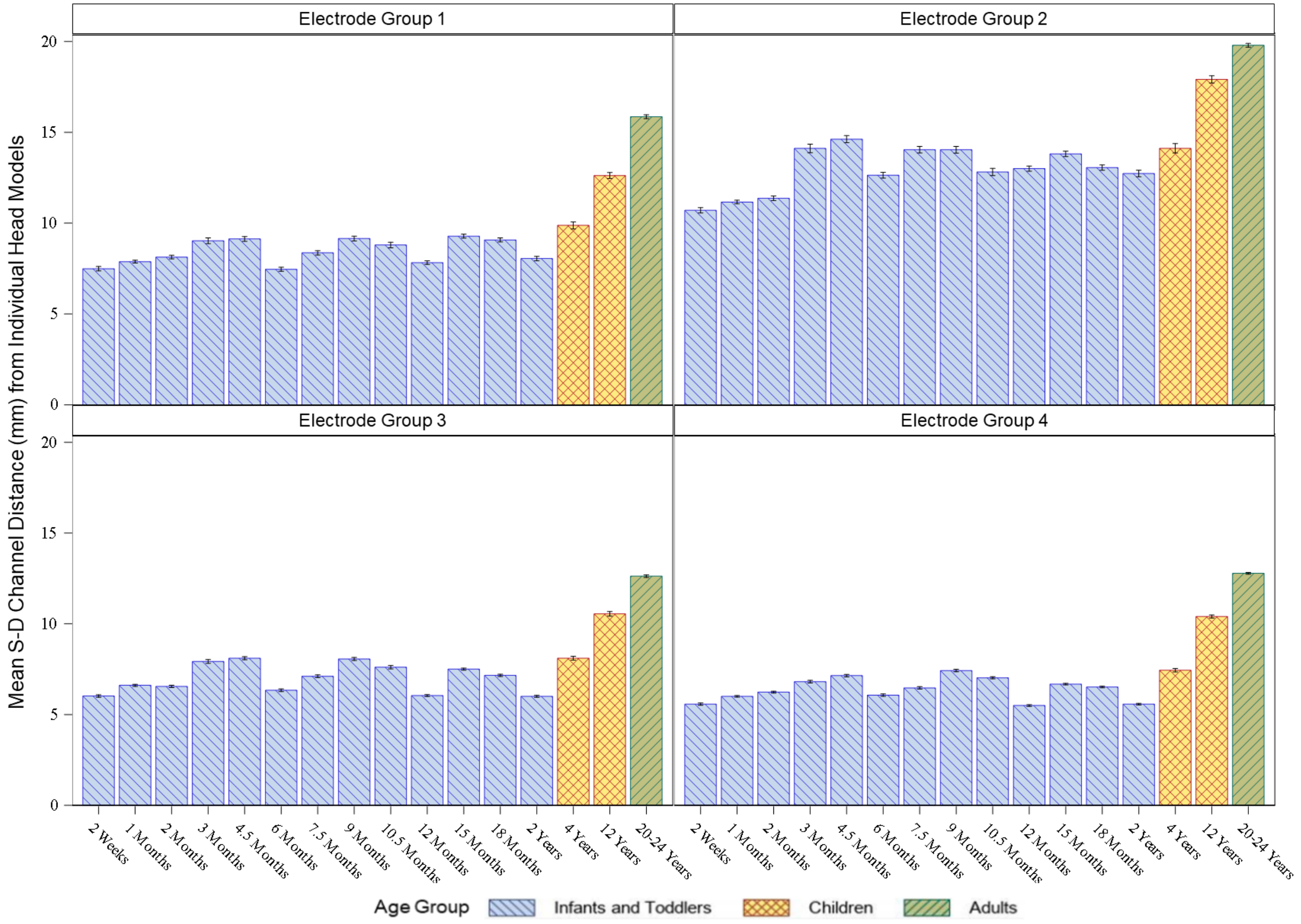

Fig S5. Mean Source-Detector (S-D) Channel DOT distances by age group and electrode group. A. Mean S-D Channel DOT distances computed from age-matched averaged MRI templates. B. Mean S-D Channel DOT distances computed from individual head models (c.f. Main Text Figure 4).

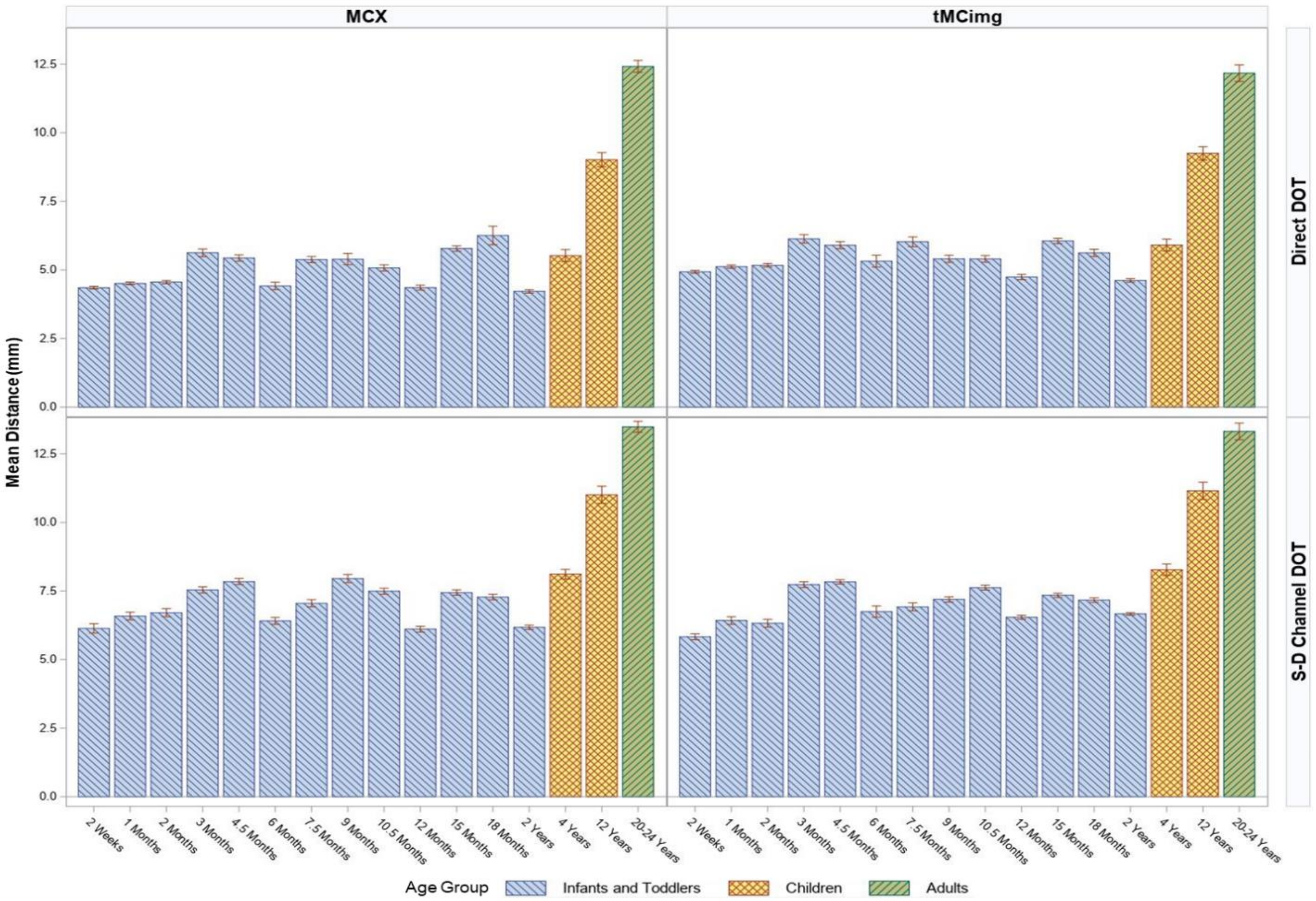

Fig S6. Comparison of Direct DOT and S-D Channel DOT distance measures from MCX and tMCimg outputs.

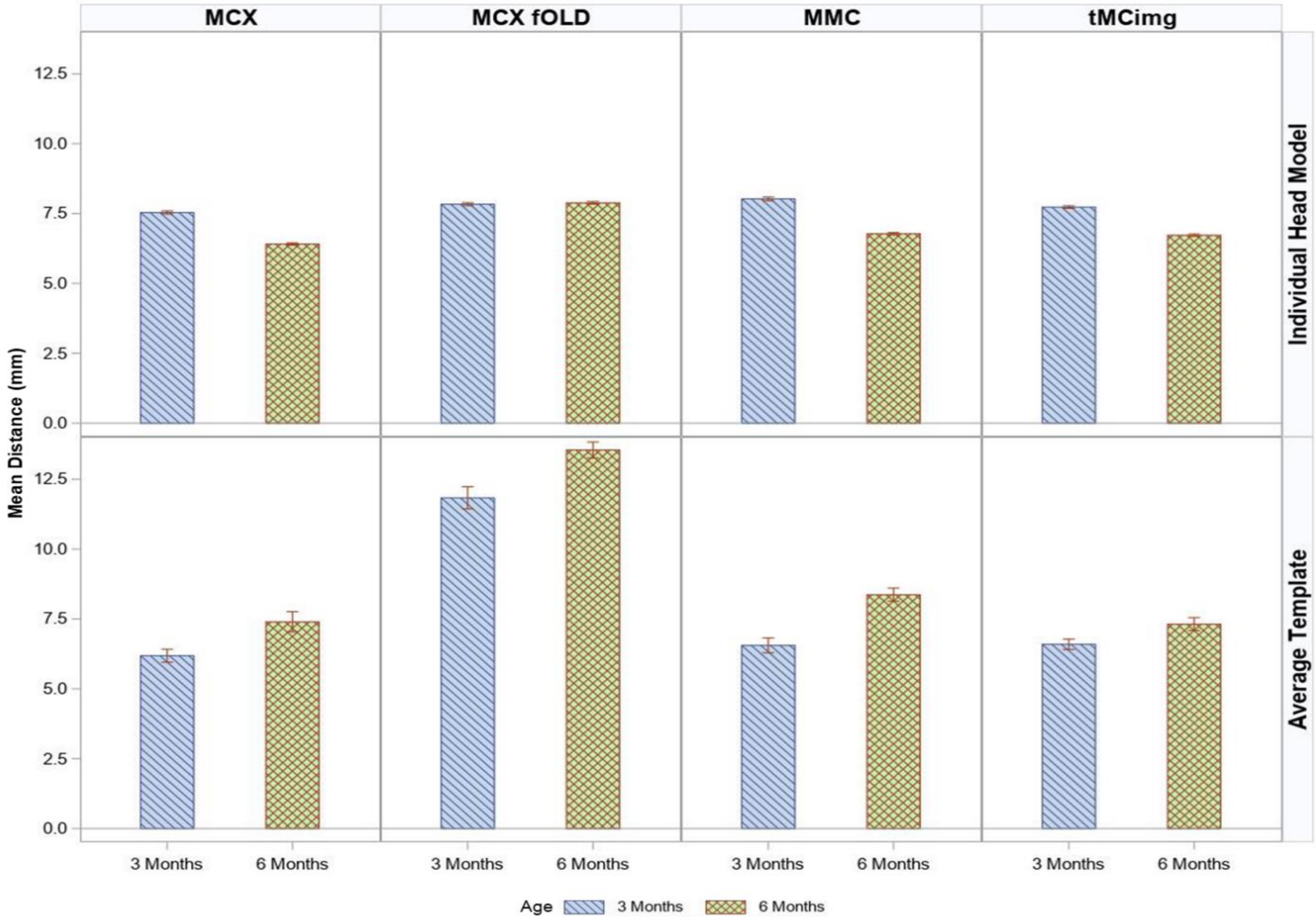

Fig S7. A comparison of S-D channel DOT distance among estimation methods (MCX, MCX with fOLD channels, MMC, tMCimg) and head model types (individual head models and age-matched average templates) in 3- and 6-month-old infants.
